# Efficient Iterative Arbitrary High-Order Methods: an Adaptive Bridge Between Low and High Order

**DOI:** 10.1007/s42967-023-00290-w

**Published:** 2023-09-11

**Authors:** Lorenzo Micalizzi, Davide Torlo, Walter Boscheri

**Affiliations:** 1 Institute of Mathematics, University of Zurich, Winterthurerstrasse 190, Zurich, 8057 Switzerland; 2 SISSA mathLab, SISSA, via Bonomea 265, Trieste, 34136 Italy; 3 Dipartimento di Matematica e Informatica, University of Ferrara, via Machiavelli 30, Ferrara, 44121 Italy

**Keywords:** *p*-Adaptivity, Arbitrary DERivative (ADER), Arbitrary high order, Deferred Correction (DeC), Positivity preserving, 65M60, 65Y20, 35L65

## Abstract

We propose a new paradigm for designing efficient *p*-adaptive arbitrary high-order methods. We consider arbitrary high-order iterative schemes that gain one order of accuracy at each iteration and we modify them to match the accuracy achieved in a specific iteration with the discretization accuracy of the same iteration. Apart from the computational advantage, the newly modified methods allow to naturally perform the *p*-adaptivity, stopping the iterations when appropriate conditions are met. Moreover, the modification is very easy to be included in an existing implementation of an arbitrary high-order iterative scheme and it does not ruin the possibility of parallelization, if this was achievable by the original method. An application to the Arbitrary DERivative (ADER) method for hyperbolic Partial Differential Equations (PDEs) is presented here. We explain how such a framework can be interpreted as an arbitrary high-order iterative scheme, by recasting it as a Deferred Correction (DeC) method, and how to easily modify it to obtain a more efficient formulation, in which a local *a posteriori* limiter can be naturally integrated leading to the *p*-adaptivity and structure-preserving properties. Finally, the novel approach is extensively tested against classical benchmarks for compressible gas dynamics to show the robustness and the computational efficiency.

## Introduction

In recent years, the need for a very accurate description of physical phenomena in the context of advanced technological applications has determined an increasing interest toward large-scale simulations. To reduce their enormous computational cost and to make them more accessible, several strategies have been proposed, among which:

- parallelization, leading to a reduction of the computational time proportional to the number of employed processors with excellent scaling properties [39, 52, 55, 68, 73, 81, 82];
- structure-preserving schemes, to preserve physical properties at the discrete level without excessive mesh refinements, e.g., positivity preserving schemes [27, 53, 60, 66, 67], well-balanced schemes [12, 22, 25–28, 47, 59, 64, 85], TVD or maximum principle preserving schemes [10, 48, 49, 86], and entropy conservative/dissipating schemes [4–7, 23, 24, 40, 44, 45, 56–58, 65, 69, 83, 84];
- high-order methods, which guarantee higher accuracy for coarser meshes and shorter computational times, on smooth problems, as they are able to catch complicated physical structures that low-order methods struggle to obtain, e.g., finite-element-based methods [1, 3, 8, 54, 57, 70, 71], finite volume methods [10, 12, 22, 64, 75, 85], and discontinuous Galerkin (DG) methods [17, 19, 23, 35, 44–46, 51].

However, high-order methods are, for the moment, mostly relegated to academic contexts. The main reason is given by the fact that concrete applications are characterized by shocks, which are well known to reduce the accuracy to first order, disregarding for the formal order of accuracy. Further, in the presence of shocks, high-order schemes are more subjected to instabilities. Users are, therefore, comprehensibly unwilling to pay extra costs in terms of the complexity of the numerical method and its implementation, if the effort is not rewarded with the initially expected advantages. Indeed, one can observe that in the case of non-regular solutions; usually, the shocks do not cover the whole computational domain but rather some lower dimensional manifolds. Therefore, a possibility to use high-order methods at their best is the adoption of extra procedures to be implemented, e.g., limiters, *a posteriori* correction techniques, blenders with low-order schemes, or adaptive strategies relying on shock detectors. However, such procedures require a relevant interference with the basic implementation as they are not naturally embedded in the original method at the theoretical level and, if their introduction is not performed in a careful way, the additional cost associated with them may be comparable to the computational gain given by the high-order feature.

Here, generalizing the idea introduced in [61], we propose a new arbitrary high-order formulation naturally allowing for order adaptivity, namely the so-called *p*-adaptivity. The formulation relies on an underlying arbitrary high-order iterative scheme, which is easily modified in a suitable efficient way. Arbitrary high-order iterative methods are characterized by iterative procedures involving an approximated solution to a certain problem, whose order of accuracy increases by one at each iteration, converging toward the solution of a background high-order scheme. The idea is to modify the generic iteration in such a way that the order of accuracy of the discretization proper to the iteration itself matches the order of accuracy achieved in that specific iteration, hence reducing the computational cost. The number of iterations is chosen equal to the aimed order of accuracy, as already done in [8, 38, 50, 61, 63]. On the contrary, in other works, the iterations are stopped when a prescribed tolerance is reached [14, 21, 34, 42, 79]. This is most of the times unnecessary for explicit methods, as the accuracy of the underlying discretization is not as accurate as that tolerance.

The modification we propose in this work results in several advantages: for a fixed final order, we get a substantial drop in the computational cost with respect to the traditional approach as the low-order iterations are performed with low-order structures which are computationally cheaper. Moreover, in this framework, it is straightforward to limit the achieved order on the fly, stopping the computation at a certain iteration if specified criteria are not met. This last aspect allows to overcome the typical drawback of *a posteriori* Multi-dimensional Optimal Order Detection (MOOD) techniques [9, 17, 18, 29, 31, 32], in which, if the high-order scheme produces a solution which is not valid according to some physical or numerical criteria, low-order solutions must be recomputed with their associated computational cost after the high-order solution has been already computed.

Apart from the advantages and the many possible applications, a remarkable aspect which is worth underlying is given by the fact that, if one already has an implementation of an arbitrary high-order iterative scheme, then the introduction of the proposed modification is straightforward. Furthermore, the modification does not prevent the possible parallelization of the original code.

In this paper, we discuss the application to an Arbitrary DERivative (ADER) framework for hyperbolic Partial Differential Equations (PDEs), proposed originally by Millington et al. [62, 78]. The approach is validated on challenging benchmarks, showing the arbitrary high-order character and the optimal performance in the context of the adaptivity. Furthermore, the efficiently designed *a posteriori* limiter, which drives the *p*-adaptivity, allows to provably preserve physical properties. In this work, we will use it to preserve the positivity of some quantities associated with the numerical solution. The resulting numerical schemes are of high order of accuracy with one-step time discretization and making use of general polygonal cells in space.

Summarizing, the main contributions of this work are the following: the generalization of the idea introduced in [61] into an abstract and rigorous framework, its application to the ADER context for hyperbolic PDEs with increasing reconstruction degree along the iterations of the space-time predictor solution, and the design of an efficient structure-preserving *a posteriori* limiter.

The work is organized as follows. In Sect. 2, we describe the general idea in the specific framework of Deferred Correction (DeC) methods. In Sect. 3, we present the ADER method for hyperbolic PDEs with the DG spatial discretization and we explain how it can be interpreted as an iterative arbitrary high-order method. Section 4 is devoted to the description of the proposed modifications for the ADER framework to obtain new efficient *p*-adaptive schemes. The new methods are validated against several challenging benchmarks in Sect. 5, demonstrating the accuracy and the robustness of the novel approach. Moreover, the computational advantages with respect to the original formulation are experimentally shown. Finally, conclusions and future perspectives are reported in Sect. 6.

## New Efficient Iterative Arbitrary High-Order Methods

Iterative arbitrary high-order methods are numerical methods characterized by an iteration process converging to the solution of an underlying arbitrary high-order scheme. Here, we focus on particular iterative arbitrary high-order methods, for which the order of accuracy with respect to the limit solution increases by one at each iteration. Examples of such methods are the DeC [2, 8, 38, 50, 63] and the ADER schemes [16, 34, 35, 51], which have been broadly used in the context of the numerical solution of hyperbolic systems of PDEs. The current use of such methods consists in fixing an underlying high-order scheme and performing the iteration process until convergence or, more efficiently, until the desired accuracy is reached, i.e., with a number of iterations exactly equal to the order of the method.

We propose here a new simple modification of the aforementioned framework which allows the computational cost of the original methods to be reduced, designing novel schemes with a natural adaptive character. In particular, let us consider a general iterative arbitrary high-order method of order *P*, whose generic iteration is denoted by ${\mathcal {M}}_P$. Then, a simple sketch of the method is given by

1 $$\begin{aligned} \underline{\varvec{u}}^{(0)} \xrightarrow {{\mathcal {M}}_P} \underline{\varvec{u}}^{(1)} \xrightarrow {{\mathcal {M}}_P} \underline{\varvec{u}}^{(2)} \xrightarrow {{\mathcal {M}}_P} \cdots \xrightarrow {{\mathcal {M}}_P} \underline{\varvec{u}}^{(P-1)} \xrightarrow {{\mathcal {M}}_P} \underline{\varvec{u}}^{(P)}, \end{aligned}$$

where $\underline{\varvec{u}}^{(p)}$ is the result of the *p*th iteration and *P* iterations have been considered to achieve the optimal accuracy with the minimal number of iterations. The proposed modification consists in replacing the generic *p*th iteration of the method of order *P* with the iteration ${\mathcal {M}}_p$, that is the iteration associated to the same method but with order *p* which is in general cheaper but, nevertheless, accurate enough to get order *p*. The sketch of the modified method reads

2 $$\begin{aligned} \underline{\varvec{u}}^{(0)} \xrightarrow {{\mathcal {M}}_1} \underline{\varvec{u}}^{(1)} \xrightarrow {{\mathcal {M}}_2} \underline{\varvec{u}}^{(2)} \xrightarrow {{\mathcal {M}}_{3}} \cdots \xrightarrow {{\mathcal {M}}_{P-1}} \underline{\varvec{u}}^{(P-1)} \xrightarrow {{\mathcal {M}}_P} \underline{\varvec{u}}^{(P)}. \end{aligned}$$

The formal order of accuracy is not spoiled as ${\mathcal {M}}_p$, the new *p*th iteration, is still sufficiently accurate to provide $\underline{\varvec{u}}^{(p)}$ of order *p* starting by $\underline{\varvec{u}}^{(p-1)}$ of order $p-1$. The technical details of changing the iteration structures at each iteration depend on the underlying iterative arbitrary high-order method under consideration. For example, an intermediate embedding process like an interpolation may be needed to project $\underline{\varvec{u}}^{(p-1)}$ onto the same space of $\underline{\varvec{u}}^{(p)}$ in order to perform the *p*th iteration. The modification to pass from the original formulation (1) to the new one (2) is minimal, and its inclusion in an existing implementation of an iterative arbitrary high-order method is straightforward. The modified methods are in general cheaper than the original ones, as the computational cost related to ${\mathcal {M}}_p$ for $p<P$ is smaller than the one related to ${\mathcal {M}}_P$. Moreover, differently from what happens in the original framework, increasing the number of iterations always determines an increase in the order of accuracy without any saturation. Therefore, in principle, it is possible not to fix *a priori* the final order, but instead to continue the iterations until a certain tolerance is matched. This may provide a valid strategy for engineering applications.

We focus now on a particular family of iterative arbitrary high-order methods, and we discuss their modification to comply with the *p*-adaptivity setting proposed in this work.

### DeC Methods

The DeC is an abstract procedure that can be exploited to design arbitrary high-order iterative methods for differential problems. In particular, the formulation presented in [2] relies on the definition of two operators $\mathcal {L}_{\Delta }^1,\mathcal {L}_{\Delta }^2 : X \rightarrow Y$ associated with a given problem, dependent on a parameter $\Delta$, acting between two normed vector spaces $(X,\left\Vert \cdot \right\Vert _X)$ and $(Y,\left\Vert \cdot \right\Vert _Y)$. The operator $\mathcal {L}_{\Delta }^2$ is a high-order nonlinear implicit operator that we would like to solve, i.e., to find $\underline{\varvec{u}}_\Delta \in X$, such that $\mathcal {L}_{\Delta }^2(\underline{\varvec{u}}_\Delta )=\varvec{0}_Y$, to get a high-order approximation of the solution to the original problem. Nevertheless, due to its implicit nature, the operator $\mathcal {L}_{\Delta }^2$ is difficult to be solved. On the other hand, the operator $\mathcal {L}_{\Delta }^1$ is a low-order explicit operator, for which it is easy to find ${\widetilde{\underline{\varvec{u}}}}\in X$, such that $\mathcal {L}_{\Delta }^1({\widetilde{\underline{\varvec{u}}}})=\underline{\varvec{z}}$ for $\underline{\varvec{z}}\in Y.$ Due to its simplicity, it would be desirable to solve $\mathcal {L}_{\Delta }^1$, rather than $\mathcal {L}_{\Delta }^2$. However, the resulting solution would not be accurate enough for our purposes.

The following theorem allows to approximate $\underline{\varvec{u}}_\Delta$ arbitrarily well by a simple explicit iterative procedure, which is much cheaper than the direct solution of the operator $\mathcal {L}_{\Delta }^2$.

#### Theorem 1

(DeC) Let the operators $\mathcal {L}_{\Delta }^1$ and $\mathcal {L}_{\Delta }^2$ fulfill the following properties.

(i) *Existence of a unique solution to* $\mathcal {L}_\Delta ^2$
  $\exists ! \,\underline{\varvec{u}}_\Delta \in X$ solution of $\mathcal {L}_\Delta ^2$, such that $\mathcal {L}_\Delta ^2(\underline{\varvec{u}}_\Delta )=\varvec{0}_Y$.
(ii) *Coercivity-like property of* $\mathcal {L}_\Delta ^1$
  $$\exists \,\alpha _1 \geqslant 0$$ independent of $$\Delta$$ s.t.
  3 $$\begin{aligned} \left\Vert \mathcal {L}_\Delta ^1(\underline{\varvec{v}})-\mathcal {L}_\Delta ^1(\underline{\varvec{w}})\right\Vert _Y\geqslant \alpha _1\left\Vert \underline{\varvec{v}}-\underline{\varvec{w}}\right\Vert _X, ~ \forall \underline{\varvec{v}},\underline{\varvec{w}}\in X. \end{aligned}$$
(iii) *Lipschitz-continuity-like property of* $\mathcal {L}_\Delta ^1-\mathcal {L}_\Delta ^2$
  $$\exists \, \alpha _2 \geqslant 0$$ independent of $$\Delta$$ s.t.
  4 $$\begin{aligned} \left\Vert \left[ \mathcal {L}_\Delta ^1(\underline{\varvec{v}})\!-\!\mathcal {L}_\Delta ^2(\underline{\varvec{v}})\right] \!-\! \left[ \mathcal {L}_\Delta ^1(\underline{\varvec{w}})\!-\!\mathcal {L}_\Delta ^2(\underline{\varvec{w}})\right] \right\Vert _Y\!\leqslant \!\alpha _2 \Delta \!\left\Vert \underline{\varvec{v}}-\underline{\varvec{w}}\right\Vert _X,~\forall \underline{\varvec{v}},\underline{\varvec{w}}\in X. \end{aligned}$$

*Given a* $\underline{\varvec{u}}^{(0)}\in X$, *define recursively the sequence of vectors* $\underline{\varvec{u}}^{(p)}$ *as the solution of*

5 $$\begin{aligned} \mathcal {L}_\Delta ^1(\underline{\varvec{u}}^{(p)}):=\mathcal {L}_\Delta ^1(\underline{\varvec{u}}^{(p-1)})-\mathcal {L}_\Delta ^2(\underline{\varvec{u}}^{(p-1)}), \quad p\geqslant 1. \end{aligned}$$

Then, the following error estimate holds:

6 $$\begin{aligned} \left\Vert \underline{\varvec{u}}^{(p)}-\underline{\varvec{u}}_\Delta \right\Vert _X \leqslant \left( \Delta \frac{\alpha _2}{\alpha _1} \right) ^p\left\Vert \underline{\varvec{u}}^{(0)}-\underline{\varvec{u}}_\Delta \right\Vert _X, \quad \forall p\in {\mathbb {N}}. \end{aligned}$$

#### Proof

Using the coercivity-like property of $\mathcal {L}_\Delta ^1$ (3), the definition of the DeC iteration (5), the fact that $\mathcal {L}_\Delta ^2(\underline{\varvec{u}}_\Delta )=\varvec{0}_Y$, and the Lipschitz-continuity-like property of $\mathcal {L}_\Delta ^1-\mathcal {L}_\Delta ^2$ (4), we get

7 $$\begin{aligned} \begin{aligned} \left\Vert \underline{\varvec{u}}^{(p)}-\underline{\varvec{u}}_\Delta \right\Vert _X&\leqslant \frac{1}{\alpha _1} \left\Vert \mathcal {L}_\Delta ^1(\underline{\varvec{u}}^{(p)})-\mathcal {L}_\Delta ^1(\underline{\varvec{u}}_\Delta )\right\Vert _Y\\&= \frac{1}{\alpha _1} \left\Vert \mathcal {L}_\Delta ^1(\underline{\varvec{u}}^{(p-1)})-\mathcal {L}_\Delta ^2 (\underline{\varvec{u}}^{(p-1)})-\mathcal {L}_\Delta ^1(\underline{\varvec{u}}_\Delta )\right\Vert _Y\\&= \frac{1}{\alpha _1} \left\Vert \left[ \mathcal {L}_\Delta ^1(\underline{\varvec{u}}^{(p-1)})-\mathcal {L}_\Delta ^2 (\underline{\varvec{u}}^{(p-1)})\right] -\left[ \mathcal {L}_\Delta ^1(\underline{\varvec{u}}_\Delta )-\mathcal {L}_\Delta ^2(\underline{\varvec{u}}_\Delta )\right] \right\Vert _Y\\&\leqslant \Delta \frac{\alpha _2}{\alpha _1} \left\Vert \underline{\varvec{u}}^{(p-1)}-\underline{\varvec{u}}_\Delta \right\Vert _X. \end{aligned} \end{aligned}$$

Applying recursively the previous inequality, we obtain the desired result.

For $\Delta$ small enough, the sequence of vectors $\underline{\varvec{u}}^{(p)}$ converges to $\underline{\varvec{u}}_\Delta$ independently of the initial vector $\underline{\varvec{u}}^{(0)}.$ At each iteration of the DeC procedure (5), the computation of $\underline{\varvec{u}}^{(p)}$ is straightforward by our assumptions on the operator $\mathcal {L}_{\Delta }^1$, since $\underline{\varvec{u}}^{(p-1)}$ is known and it is possible to explicitly compute the right-hand side. Furthermore, thanks to the accuracy estimate (6), at each iteration, one order of accuracy is gained with respect to $\underline{\varvec{u}}_\Delta$.

#### Remark 1

(On “over-resolving” the operator $\mathcal {L}_{\Delta }^2$ and on the number of iterations *P*) Usually, we are not strictly interested in the solution $\underline{\varvec{u}}_\Delta$ of the operator $\mathcal {L}_{\Delta }^2$, but rather on the analytical solution $\underline{\varvec{u}}_{\text{ex}}$ of the underlying problem. If *S* is the order of accuracy of $\underline{\varvec{u}}_\Delta$, in general, it suffices to approximate $\underline{\varvec{u}}_\Delta$ with the *S*th-order accuracy. This consideration allows to bound the number of iterations, saving computational time, without necessarily getting the convergence toward the solution to the operator $\mathcal {L}_{\Delta }^2$. In particular, thanks to Theorem 1, if $\underline{\varvec{u}}^{(0)}$ is an $O(\Delta )$-approximation of $\underline{\varvec{u}}_{\text{ex}}$, then $\left\Vert \underline{\varvec{u}}^{(P)}-\underline{\varvec{u}}_{\text{ex}}\right\Vert = O(\Delta ^{1+\min {(P,S)}})$ leading to an order of accuracy equal to $\min {(P,S)}$. Hence, the optimal choice is $P=S$. Any further iteration will not increase the order of accuracy of the method with respect to $\underline{\varvec{u}}_{\text{ex}}$ but only with respect to $\underline{\varvec{u}}_\Delta$.

#### Remark 2

(On explicit and implicit DeC methods) The DeC philosophy is based on having a simple iterative procedure allowing to obtain a high-order approximation of the solution of a given problem, which would have been difficult to compute directly. In the applications, we will focus on an explicit setting by considering explicit operators $\mathcal {L}_{\Delta }^1$, leading to an updated formula (5) with an explicit character. However, within the described framework, one could easily switch to an implicit setting by selecting an implicit low-order operator $\mathcal {L}_{\Delta }^1$. In that case, one obtains an iterative procedure that is not explicit, yet, much simpler than the direct solution of $\mathcal {L}_{\Delta }^2$. In applications to ordinary differential equations (ODEs) and PDEs, implicit formulations [8, 17, 20] allow to achieve better stability properties and less time step restrictions. The theoretical framework presented in this work applies both to explicit and implicit settings.

### New Efficient DeC Methods

Here, we will discuss, at the theoretical level, an efficient modification for DeC methods. It is based on the replacement of the operators $\mathcal {L}_{\Delta }^{1}$ and $\mathcal {L}_{\Delta }^{2}$ by iteration-specific operators $\mathcal {L}_{\Delta }^{1,(p)}$ and $\mathcal {L}_{\Delta }^{2,(p)}$ to strictly obtain the order *p* at the *p*th iteration. In particular, we prove the following result.

#### Theorem 2

Consider a problem with an exact solution $\underline{\varvec{u}}_{\text{ex}}\in Z$. Then, take some normed spaces $(X^{(p)},\left\Vert \cdot \right\Vert _{X^{(p)}})$ for $p\in {\mathbb {N}}$ and $(Y^{(p)},\left\Vert \cdot \right\Vert _{Y^{(p)}})$ for $p\geqslant 1$. For every $p \geqslant 1$, consider also two operators $\mathcal {L}_{\Delta }^{1,(p)},\mathcal {L}_{\Delta }^{2,(p)} : X^{(p)}\rightarrow Y^{(p)}$ dependent on the same parameter $\Delta$ and fulfilling the properties of Theorem 1 for some $\alpha _{1}^{(p)}, \alpha _{2}^{(p)}>0$ and $\underline{\varvec{u}}_{\Delta }^{(p)}\in X^{(p)}$. Furthermore, let us assume that for any $p\in {\mathbb {N}}$, there exists an embedding operator $\mathcal {E}^{(p)} : X^{(p)}\rightarrow X^{(p+1)}$, associating with each $\underline{\varvec{u}}^{(p)} \in X^{(p)}$ an approximation $\underline{\varvec{u}}^{*(p)}:=\mathcal {E}^{(p)}(\underline{\varvec{u}}^{(p)}) \in X^{(p+1)}$, and some projection $\Pi ^{(p)} : Z\rightarrow X^{(p)}$, associating with $\underline{\varvec{u}}_{\text{ex}}$ an approximation $\underline{\varvec{u}}_{\text{ex}}^{(p)}{:}=\Pi ^{(p)}(\underline{\varvec{u}}_{\text{ex}}) \in X^{(p)}.$

Given $\underline{\varvec{u}}^{(0)}\in X^{(0)}$, we consider the new DeC method whose general *p*th iteration is given by

8 $$\begin{aligned} {\left\{ \begin{array}{ll} \underline{\varvec{u}}^{*(p-1)}:={\mathcal {E}}^{(p-1)}(\underline{\varvec{u}}^{(p-1)}),\\ \mathcal {L}_\Delta ^{1,(p)}(\underline{\varvec{u}}^{(p)}):=\mathcal {L}_\Delta ^{1,(p)} (\underline{\varvec{u}}^{*(p-1)})-\mathcal {L}_\Delta ^{2,(p)}(\underline{\varvec{u}}^{*(p-1)}). \end{array}\right. } \end{aligned}$$

Suppose that the following properties hold:

(i) *the accuracy of* $\underline{\varvec{u}}_{\Delta }^{(p)}$ *with respect to* $\underline{\varvec{u}}_{\text{ex}}^{(p)}$
  9 $$\begin{aligned} \left\Vert \underline{\varvec{u}}_{\Delta }^{(p)}-\underline{\varvec{u}}_{\text{ex}}^{(p)}\right\Vert _{X^{(p)}}=O(\Delta ^{p+1}), \quad p \geqslant 1; \end{aligned}$$
(ii) *the accuracy of the embedding* $\mathcal {E}^{(p)}$
  10 $$\begin{aligned} \left\Vert \underline{\varvec{u}}^{*(p)}-\underline{\varvec{u}}_{\text{ex}}^{(p+1)}\right\Vert _{X^{(p+1)}}\leqslant C\left\Vert \underline{\varvec{u}}^{(p)}-\underline{\varvec{u}}_{\text{ex}}^{(p)}\right\Vert _{X^{(p)}}, \quad \forall p\in {\mathbb {N}} \end{aligned}$$
  *for some constant C* *independent on *$\Delta$;
(iii) *the accuracy of* $\underline{\varvec{u}}^{(0)}$
  11 $$\begin{aligned} \left\Vert \underline{\varvec{u}}^{(0)}-\underline{\varvec{u}}_{\text{ex}}^{(0)}\right\Vert _{X^{(0)}}=O(\Delta ). \end{aligned}$$

*Then*, *it follows that*

12 $$\begin{aligned} \left\Vert \underline{\varvec{u}}^{(p)}-\underline{\varvec{u}}_{\text{ex}}^{(p)}\right\Vert _{X^{(p)}}=O(\Delta ^{p+1}), \quad \forall p\in {\mathbb {N}}. \end{aligned}$$

#### Proof

The proof is based on the induction. The base case for $p=0$ is trivially given by assumption (11). Let us now focus on the induction step. We assume that (12) holds for a specific *p* and we will prove it for $p+1$. By the triangular inequality, we have

13 $$\begin{aligned} \left\Vert \underline{\varvec{u}}^{(p+1)}-\underline{\varvec{u}}_{\text{ex}}^{(p+1)}\right\Vert _{X^{(p+1)}}\leqslant \left\Vert \underline{\varvec{u}}^{(p+1)}-\underline{\varvec{u}}_{\Delta }^{(p+1)}\right\Vert _{X^{(p+1)}}+ \left\Vert \underline{\varvec{u}}_{\Delta }^{(p+1)}-\underline{\varvec{u}}_{\text{ex}}^{(p+1)}\right\Vert _{X^{(p+1)}}. \end{aligned}$$

The second term at the right-hand side is $O(\Delta ^{p+2})$ for (9), and hence, let us focus on the first term. By the proof of Theorem 1 concerning the original methods, we have that

14 $$\begin{aligned} \left\Vert \underline{\varvec{u}}^{(p+1)}-\underline{\varvec{u}}_{\Delta }^{(p+1)}\right\Vert _{X^{(p+1)}}\leqslant \Delta \frac{\alpha _{2}^{(p+1)}}{\alpha _{1}^{(p+1)}}\left\Vert \underline{\varvec{u}}^{*(p)}-\underline{\varvec{u}}_{\Delta }^{(p+1)}\right\Vert _{X^{(p+1)}}, \end{aligned}$$

which, applying the triangular inequality, gives

15 $$\begin{aligned} \begin{aligned}&\left\Vert \underline{\varvec{u}}^{(p+1)}-\underline{\varvec{u}}_{\Delta }^{(p+1)}\right\Vert _{X^{(p+1)}} \\ \leqslant &\,\Delta \frac{\alpha _{2}^{(p+1)}}{\alpha _{1}^{(p+1)}}\left( \left\Vert \underline{\varvec{u}}^{*(p)}-\underline{\varvec{u}}_{\text{ex}}^{(p+1)} \right\Vert _{X^{(p+1)}}+\left\Vert \underline{\varvec{u}}_{\text{ex}}^{(p+1)}-\underline{\varvec{u}}_{\Delta }^{(p+1)}\right\Vert _{X^{(p+1)}} \right) . \end{aligned} \end{aligned}$$

Again, due to (9), the second term in parenthesis at the right-hand side is $O(\Delta ^{p+2})$; therefore, we focus on the first term. Due to the assumption on the accuracy of the embedding (10) and to the induction hypothesis, we have

16 $$\begin{aligned} \left\Vert \underline{\varvec{u}}^{*(p)}-\underline{\varvec{u}}_{\text{ex}}^{(p+1)} \right\Vert _{X^{(p+1)}}\leqslant C\left\Vert \underline{\varvec{u}}^{(p)}-\underline{\varvec{u}}_{\text{ex}}^{(p)}\right\Vert _{X^{(p)}} = O(\Delta ^{p+1}). \end{aligned}$$

Hence

17 $$\begin{aligned} \Delta \frac{\alpha _{2}^{(p+1)}}{\alpha _{1}^{(p+1)}}\left\Vert \underline{\varvec{u}}^{*(p)}-\underline{\varvec{u}}_{\text{ex}}^{(p+1)} \right\Vert _{X^{(p+1)}}=O(\Delta ^{p+2}), \end{aligned}$$

which completes the proof.

Let us notice that, in the previous theorem, the accuracy estimate is always referred to a projection of the exact solution and not to a fixed high-order approximation. Hence, the order of accuracy is formally not bounded and we can approximate $\underline{\varvec{u}}_{\text{ex}}$ arbitrarily well. In particular, if $\underline{\varvec{u}}_{\text{ex}}^{(p)}$ yields an approximation of $\underline{\varvec{u}}_{\text{ex}}$ which is $O(\Delta ^{p+1})$ accurate, thanks to Theorem 2, also the approximation associated with $\underline{\varvec{u}}^{(p)}$ will have the same accuracy with respect to $\underline{\varvec{u}}_{\text{ex}}$.

## ADER-Discontinuous Galerkin (DG) Scheme

The ADER methods are various techniques to obtain arbitrary high-order methods for differential problems. Even though the first ADER [82] was based on the Cauchy-Kovalevskaya theorem; nowadays, it is mainly known as a technique that exploits the weak formulation of the original problem to obtain high-order discretization forms that are solved iteratively [16, 34, 50]. In this section, we will present a formulation for hyperbolic PDEs in combination with a DG space discretization, and we will show how it can be interpreted as an arbitrary high-order iterative method in the previously presented DeC framework. We will also describe in a final subsection the ADER-$\mathbb P_N \mathbb P_M$ variant of the method, still recastable as the DeC scheme, which allows for applications to finite volume (FV) formulations, as well.

### Numerical Method

We want to approximate the analytical solution $\varvec{u} : {\overline{\varOmega }} \times {\mathbb {R}}^+_0 \rightarrow {\mathbb {R}}^Q$ of the following *Q*-dimensional hyperbolic PDE:

18 $$\begin{aligned} \frac{\partial }{\partial t}\varvec{u}(\varvec{x},t)+\textrm{div}_{\varvec{x}} {\varvec{F}}(\varvec{u}(\varvec{x},t))={\varvec{S}}(\varvec{x},\varvec{u}(\varvec{x},t)), \quad (\varvec{x},t) \in \varOmega \times {\mathbb {R}}^+_0, \end{aligned}$$

supplemented with suitable initial and boundary conditions, where $\varOmega \subseteq {\mathbb {R}}^D$ is a bounded *D*-dimensional space domain, ${\varvec{F}} : \mathbb {R}^Q \rightarrow \mathbb {R}^{Q\times D}$ is the flux tensor, and ${\varvec{S}} : {\overline{\varOmega }} \times \mathbb {R}^{Q} \rightarrow \mathbb {R}^Q$ is the source function. To shorten the notation, let us define $\varvec{E}(\varvec{u},\varvec{x}):=\textrm{div}_{\varvec{x}} {\varvec{F}}(\varvec{u})-{\varvec{S}}(\varvec{x},\varvec{u})$, the time evolution operator of the PDE up to the minus sign, so that (18) becomes

19 $$\begin{aligned} \frac{\partial }{\partial t}\varvec{u}(\varvec{x},t)+\varvec{E}(\varvec{u}(\varvec{x},t),\varvec{x})=\varvec{0}, \quad (\varvec{x},t) \in \varOmega \times {\mathbb {R}}^+_0. \end{aligned}$$

Let us focus on a generic time step $[t_n,t_{n+1}]$ with $\Delta t:=t_{n+1}-t_n$. The goal is to find an approximation $\varvec{u}_{n+1}(\varvec{x})\approx \varvec{u}(\varvec{x},t_{n+1})$ of the analytical solution in ${\overline{\varOmega }}$ at time $t_{n+1}$ by knowing an approximation $\varvec{u}_{n}(\varvec{x})\approx \varvec{u}(\varvec{x},t_{n})$ at time $t_n.$

In particular, for any *n*, we adopt a classical DG space discretization for $\varvec{u}_{n}(\varvec{x})\approx \varvec{u}(\varvec{x},t_{n})$: we consider a tessellation $\mathcal {T}_h$ of ${\overline{\varOmega }}$ made of non-overlapping convex polytopals *K* with a mesh parameter *h*, and we consider $\varvec{u}_n(\varvec{x})$ in a space of discontinuous piecewise polynomial functions of degree *M*, i.e., $(V_M)^Q$ with $V_M:=\left\{ g \in L^2(\varOmega ) ~\text {s.t.}~ g\vert _K \in {\mathbb {P}}_M(K)\right\}$, yielding an $(M+1)$th order of accuracy approximation space. Therefore, locally in each element *K*, we can consider the following representation of the approximated solution with a local basis $\left\{ \varphi _{i}(\varvec{x}) \right\} _{i=1,\cdots ,I}$ of ${\mathbb {P}}_M(K)$:

20 $$\begin{aligned} \varvec{u}_n(\varvec{x}):=\sum _{i=1}^I \varvec{c}_{i}^n \varphi _{i}(\varvec{x}),\quad \forall \varvec{x}\in K, \end{aligned}$$

where *I* is the number of local basis functions and the label *K* on the coefficients $\varvec{c}_{i}^n$, and on the basis functions, $\varphi _{i}$ is omitted to lighten the notation as we will consider computations in a single generic element, in the sequel.

The ADER method, applied to this context, is based on the weak formulation of the governing equations (19) in space-time and it is characterized by two steps: an iterative local space-time predictor and a final corrector step, described hereafter. Before entering the details, it is useful to briefly describe the role of such steps for ADER methods. The predictor, based on a local explicit iterative procedure, is used to compute local high-order polynomial approximations of the solution in space-time control volumes without considering any communication between different cells. The solution obtained in this step is high-order accurate but not stable, as no upwinding has been taken into account in its computation. This prediction is later used in the corrector step to provide a global explicit update of the numerical solution, allowing communication between neighboring cells through numerical fluxes, which introduce the necessary upwinding and numerical dissipation to achieve the stability. A stability study of the method can be found in [34].

#### Local Space-Time Predictor

The purpose of this step is to find a high-order approximation of the solution in each space-time control volume $C_K=K\times [t_n,t_{n+1}]$. In this step, no communication between the cells happens. We consider the weak formulation of (18) over $C_K$, obtained through the multiplication by a smooth test function $\vartheta {:} C_K \rightarrow {\mathbb {R}}$, the integration over $C_K$ and subsequent integration by parts in time:

21 $$\begin{aligned} \begin{aligned}&\int _K\!\left[ \varvec{u}(\varvec{x},t_{n+1})\vartheta (\varvec{x},t_{n+1}) \!-\!\varvec{u}(\varvec{x},t_n)\vartheta (\varvec{x},t_{n})\right] \text{d}\varvec{x}-\!\! \int _{C_K}\!\!\!\!\! \varvec{u}(\varvec{x},t) \frac{\partial }{\partial t} \vartheta (\varvec{x},t) \text{d}\varvec{x}\text{d}t\\&+ \int _{C_K} \varvec{E}(\varvec{u}(\varvec{x},t),\varvec{x})\vartheta (\varvec{x},t)\, \text{d}\varvec{x}\text{d}t=\varvec{0}. \end{aligned} \end{aligned}$$

Next, we project it onto a finite-dimensional space spanned by the tensor product of the previously introduced local spatial basis $\left\{ \varphi _{i}(\varvec{x}) \right\} _{i=1,\cdots ,I}$ and a temporal basis $\left\{ \psi ^{m}(t) \right\} _{m=0,\cdots ,M}$ over $[t_{n},t_{n+1}]$ guaranteeing $(M+1)$th order of accuracy. As an example for the latter, one can think to a Lagrangian basis of degree *M* or a truncated Taylor series up to the *M*th-degree term to obtain an approximation of order $M+1$. Here, we will use modal basis functions both for space and time, although such choice is not mandatory. The basis functions are explicitly described in Appendix A. As usual in the literature, we assume the basis functions to be normalized in such a way that their maximum absolute value over $C_K$ is an *O*(1).

In particular, we consider the local discretization of $\varvec{u}_h$ in $C_K$

22 $$\begin{aligned} \varvec{u}_h(\varvec{x},t):=\sum _{i=1}^I\sum _{m=0}^M \varvec{u}_i^m\varphi _{i}(\varvec{x})\psi ^{m}(t)=\sum _{\ell =1}^L \varvec{u}^{\ell }\vartheta ^{\ell }(\varvec{x},t), \quad \forall (\varvec{x},t)\in C_K, \end{aligned}$$

in which, to shorten the notation, we have denoted by $\lbrace \vartheta ^{\ell }(\varvec{x},t)\rbrace _{\ell =1,\cdots ,L}$ a permutation of the basis functions $$\lbrace \varphi _{i}(\varvec{x})\psi ^{m}(t) \rbrace _{\begin{array}{c} i=1,\dots ,I\\ m=0,\dots ,M \end{array}}$$ and by $\varvec{u}^{\ell }$ the corresponding coefficients $\varvec{u}_i^m$, where implicitly we defined a bijection that gives $\ell = \ell (i,m)$. Finally, we consider the projection of (21) on the space-time DG functional space generated by $\lbrace \vartheta ^{\ell }(\varvec{x},t) \rbrace _{\ell =1,\cdots ,L}$, that is

23 $$\begin{aligned} \begin{aligned} & \sum _{\ell =1}^L\left[ \int _K \vartheta ^{\ell }(\varvec{x},t_{n+1})\vartheta ^{j}(\varvec{x},t_{n+1}) \text{d}\varvec{x}- \int _{C_{K}} \vartheta ^{\ell }(\varvec{x},t) \frac{\partial }{\partial t} \vartheta ^{j}(\varvec{x},t) \text{d}\varvec{x}\text{d}t \right] \varvec{u}^{\ell } \\&-\int _K \varvec{u}_n(\varvec{x})\vartheta ^{j}(\varvec{x},t_{n}) \text{d}\varvec{x}+ \int _{C_{K}}\varvec{E}(\varvec{u}_h(\varvec{x},t),\varvec{x})\vartheta ^{j}(\varvec{x},t) \text{d}\varvec{x}\text{d}t=\varvec{0}\end{aligned} \end{aligned}$$

for any $j=1,\cdots ,L$. This is a nonlinear system in the unknowns $\varvec{u}^{\ell }$, whose solution yields the $(M+1)$th-order accurate approximation (22) of the analytical solution. Let us notice that $\varvec{u}_n(\varvec{x})$ in (23) is known by assumption and the related integral involving such function can thus be computed. Notice that, to obtain a fully local formulation, the divergence theorem in space has not been applied. On the other hand, the integration by parts in time has been performed to introduce a causality effect and a dependency on the initial information at time $t_{n}$.

Now, it is possible to recast each local system (23) in a matrix-vector formulation writing

24 $$\begin{aligned} B\underline{\varvec{u}}-\underline{\varvec{r}}+\widetilde{\underline{\varvec{\phi }}}(\underline{\varvec{u}})=\varvec{0}, \end{aligned}$$

where the matrix *B* and the vectors $\underline{\varvec{u}}$, $\underline{\varvec{r}}$, and $\widetilde{\underline{\varvec{\phi }}}$ are given by

25 $$\begin{aligned} \left\{ \begin{aligned} &B_{j,\ell }:=\int _K \vartheta ^{\ell }(\varvec{x},t_{n+1})\vartheta ^{j}(\varvec{x},t_{n+1}) \text{d}\varvec{x}- \int _{C_K} \vartheta ^{\ell }(\varvec{x},t) \frac{\partial }{\partial t} \vartheta ^{j}(\varvec{x},t) \text{d}\varvec{x}\text{d}t,\\ &\underline{\varvec{u}}:=\begin{pmatrix} \varvec{u}^1\\ \vdots \\ \varvec{u}^L \end{pmatrix}, \quad \underline{\varvec{r}}:=\begin{pmatrix} \int _K \varvec{u}_n(\varvec{x})\vartheta ^{1}(\varvec{x},t_n)\text{d}\varvec{x}\\ \vdots \\ \int _K \varvec{u}_n(\varvec{x})\vartheta ^{L}(\varvec{x},t_n)\text{d}\varvec{x}\end{pmatrix}, \quad \widetilde{\underline{\varvec{\phi }}}(\underline{\varvec{u}}):= \begin{pmatrix} \int _{t_n}^{t_{n+1}} \varvec{\phi }_1(\underline{\varvec{u}},t) \text{d}t\\ \vdots \\ \int _{t_n}^{t_{n+1}} \varvec{\phi }_L(\underline{\varvec{u}},t) \text{d}t \end{pmatrix} \end{aligned} \right. \end{aligned}$$

with $\varvec{\phi }_j(\underline{\varvec{u}},t):=\int _K\varvec{E}(\varvec{u}_h(\varvec{x},t),\varvec{x})\vartheta ^{j}(\varvec{x},t) \text{d}\varvec{x}$. Let us observe that $\underline{\varvec{r}}$ is constant and explicitly computable as $\varvec{u}_n(\varvec{x})$ is known.

##### Remark 3

(On the matrix *B*) The definition of the matrix *B* is referred to a scalar PDE; it must be block-expanded for a vectorial problem. Let us notice that the elements of the matrix *B* are $O(h^D)$ due to the integral over *K* and to the normalization assumed on the basis functions. The integral in time on the second term of $B_{j, \ell }$ is balanced by the derivative in time on $\vartheta ^{j}.$

Concerning the well-posedness and the solution of the nonlinear system (24), we can prove that for $\Delta t$ small enough, it admits a unique solution which can be obtained through the iterative procedure

26 $$\begin{aligned} \underline{\varvec{u}}^{(p)}=B^{-1}\left[ \underline{\varvec{r}}-\widetilde{\underline{\varvec{\phi }}}\left( \underline{\varvec{u}}^{(p-1)} \right) \right] , \end{aligned}$$

which converges unconditionally to the solution of the system, for any initial vector $\underline{\varvec{u}}^{(0)}$. To do that, let us first prove the following useful lemma.

##### Lemma 1

(Lipschitz-continuity-like property of $\widetilde{\underline{\varvec{\phi }}}$) Under smoothness assumptions, the function $\widetilde{\underline{\varvec{\phi }}}(\cdot )$ is such that

27 $$\begin{aligned} \left\Vert \widetilde{\underline{\varvec{\phi }}}(\underline{\varvec{v}})-\widetilde{\underline{\varvec{\phi }}}(\underline{\varvec{w}}) \right\Vert _\infty \leqslant \Delta t \left| K \right| C_{\text{Lip}} \left\Vert \underline{\varvec{v}}-\underline{\varvec{w}}\right\Vert _\infty , \end{aligned}$$

where $\left\Vert \cdot \right\Vert _{\infty }$ is the infinity norm over ${\mathbb {R}}^{L\times Q}$ and $C_{\text{Lip}}$ is a constant independent of $\Delta t$ and of the element *K*.

##### Proof

By a direct computation of the generic *j*th component of the left-hand side of (27), recalling the definition of the functions $\varvec{\phi }_j$, through basic analysis, we get

28 $$\begin{aligned} \begin{aligned} &\Bigg \Vert  \int _{t_n}^{t_{n+1}} \varvec{\phi }_j(\underline{\varvec{v}},t)\text{d}t -\!\!\int _{t_n}^{t_{n+1}} \varvec{\phi }_j(\underline{\varvec{w}},t) \text{d}t \Bigg \Vert _{\infty ,Q} \!\!\!\!\leqslant \int _{t_n}^{t_{n+1}} \Big \Vert \varvec{\phi }_j(\underline{\varvec{v}},t) - \varvec{\phi }_j(\underline{\varvec{w}},t) \Big \Vert _{\infty ,Q} \!\!\! \text{d}t\\  \leqslant  & \int _{C_K}  \Big \Vert \varvec{E}(\varvec{v}_h(\varvec{x},t),\varvec{x}) -\varvec{E}(\varvec{w}_h(\varvec{x},t),\varvec{x}) \Big \Vert _{\infty ,Q} \Big \vert \vartheta ^{j}(\varvec{x},t) \Big \vert \text{d}\varvec{x}\text{d}t, \end{aligned} \end{aligned}$$

where $\left\Vert \cdot \right\Vert _{\infty ,Q}$ is the infinity norm over ${\mathbb {R}}^Q$, $\varvec{v}_h(\varvec{x},t):=\sum _{\ell =1}^L \varvec{v}^{\ell }\vartheta ^{\ell }(\varvec{x},t),$ and $\varvec{w}_h(\varvec{x},t):=\sum _{\ell =1}^L \varvec{w}^{\ell }\vartheta ^{\ell }(\varvec{x},t)\, \text{for any}\, (\varvec{x},t)\in C_K.$ For regular data, we can assume that the following Lipschitz-continuity property holds:

29 $$\begin{aligned} \Big \Vert \varvec{E}(\varvec{v}_h(\varvec{x},t),\varvec{x}) -\varvec{E}(\varvec{w}_h(\varvec{x},t),\varvec{x}) \Big \Vert _{\infty ,Q} \leqslant C_{0} \left\Vert \underline{\varvec{v}}-\underline{\varvec{w}}\right\Vert _\infty , \end{aligned}$$

where $C_0$ is a constant independent of $\Delta t$ and of the element *K*, leading to

30 $$\begin{aligned} \begin{aligned}&\Bigg \Vert \int _{t_n}^{t_{n+1}}\!\! \varvec{\phi }_j(\underline{\varvec{v}},t)\text{d}t -\!\!\int _{t_n}^{t_{n+1}}\!\! \varvec{\phi }_j(\underline{\varvec{w}},t) \text{d}t \Bigg \Vert _{\infty ,Q} \!\!\!\!\leqslant C_{0} \left\Vert \underline{\varvec{v}}-\underline{\varvec{w}}\right\Vert _\infty \int _{C_K} \Big \vert \vartheta ^{j}(\varvec{x},t) \Big \vert \text{d}\varvec{x}\text{d}t. \end{aligned} \end{aligned}$$

The space-time basis functions $\vartheta ^{j}$ are bounded in absolute value by a constant $C_\vartheta$ independent of $\Delta t$ and *K*, yielding

31 $$\begin{aligned} \begin{aligned}&\Bigg \Vert \int _{t_n}^{t_{n+1}} \varvec{\phi }_j(\underline{\varvec{v}},t)\text{d}t -\int _{t_n}^{t_{n+1}} \varvec{\phi }_j(\underline{\varvec{w}},t) \text{d}t \Bigg \Vert _{\infty ,Q}\leqslant C_{0}C_\vartheta \left\Vert \underline{\varvec{v}}-\underline{\varvec{w}}\right\Vert _\infty \Delta t \left|K\right|, \end{aligned} \end{aligned}$$

which, setting $C_{\text{Lip}}:=C_{0}C_\vartheta$ and taking the maximum over $j=1,\cdots ,L$ at the left-hand side, is the thesis.

A straightforward consequence of the previous result is the following corollary.

##### Corollary 1

(Lipschitz-continuity-like property of $B^{-1}\widetilde{\underline{\varvec{\phi }}}$) Under the assumptions of the previous lemma, it holds

32 $$\begin{aligned} \left\Vert B^{-1}\left[ \widetilde{\underline{\varvec{\phi }}}(\underline{\varvec{v}})-\widetilde{\underline{\varvec{\phi }}}(\underline{\varvec{w}})\right] \right\Vert _\infty \leqslant \Delta t {\widetilde{C}}_{\text{Lip}} \left\Vert \underline{\varvec{v}}-\underline{\varvec{w}}\right\Vert _\infty , \end{aligned}$$

where ${\widetilde{C}}_{\text{Lip}}$ is a constant independent of $\Delta t$ and of the element *K*.

##### Proof

By basic linear algebra, we have

33 $$\begin{aligned} \left\Vert B^{-1}\left[ \widetilde{\underline{\varvec{\phi }}}(\underline{\varvec{v}})-\widetilde{\underline{\varvec{\phi }}}(\underline{\varvec{w}})\right] \right\Vert _\infty \leqslant \left\Vert B^{-1}\right\Vert _\infty \left\Vert \widetilde{\underline{\varvec{\phi }}}(\underline{\varvec{v}})-\widetilde{\underline{\varvec{\phi }}}(\underline{\varvec{w}}) \right\Vert _\infty , \end{aligned}$$

where the infinity norm applied to $B^{-1}$ is the matrix norm induced by the related vector norm. As observed in Remark 3, *B* is an $O(h^D)$ and, hence, its inverse is an $O(h^{-D})$, leading to $\left\Vert B^{-1}\right\Vert _\infty \leqslant C_B h^{-D}$ for some constant $C_B$ independent of the specific element *K* and of $\Delta t$. Using this fact, in combination with the result of Lemma 1, we obtain

34 $$\begin{aligned} \left\Vert B^{-1}\left[ \widetilde{\underline{\varvec{\phi }}}(\underline{\varvec{v}})-\widetilde{\underline{\varvec{\phi }}}(\underline{\varvec{w}})\right] \right\Vert _\infty \leqslant C_B h^{-D} \Delta t \left| K \right| C_{\text{Lip}} \left\Vert \underline{\varvec{v}}-\underline{\varvec{w}}\right\Vert _\infty . \end{aligned}$$

By observing that for a regular mesh $\left|K\right|\leqslant C_\tau h^D$ for some constant $C_\tau$ independent of *K*, we get the thesis

35 $$\begin{aligned} \left\Vert B^{-1}\left[ \widetilde{\underline{\varvec{\phi }}}(\underline{\varvec{v}})-\widetilde{\underline{\varvec{\phi }}}(\underline{\varvec{w}})\right] \right\Vert _\infty&\leqslant \Delta t C_B C_\tau C_{\text{Lip}} \left\Vert \underline{\varvec{v}}-\underline{\varvec{w}}\right\Vert _\infty \end{aligned}$$

for $${\widetilde{C}}_{\text{Lip}}:=C_B C_\tau C_{\text{Lip}}.$$

This allows us to prove the existence and uniqueness of the solution of (24).

##### Proposition 1

(Well-posedness and solution of the nonlinear system) Because $\Delta t$ is small enough, the nonlinear system (24) has a unique solution, which is the limit of (26) Because $p\rightarrow +\infty$.

##### Proof

We define the map ${\mathcal {J}}:{\mathbb {R}}^{L\times Q}\rightarrow {\mathbb {R}}^{L\times Q}$ as ${\mathcal {J}}(\underline{\varvec{u}}):=B^{-1}\left[ \underline{\varvec{r}}-\widetilde{\underline{\varvec{\phi }}}( \underline{\varvec{u}}) \right] .$ It is immediate to verify that a fixed point of ${\mathcal {J}}$ (if any) is also a solution of (24) and viceversa. Due to the fact that ${\mathbb {R}}^{L\times Q}$ is finite dimensional, if we are able to prove that ${\mathcal {J}}$ is a contraction, by the Banach fixed-point theorem, we know that there exists a unique fixed point and that this can be obtained as the limit of the iterative procedure $\underline{\varvec{u}}^{(p)}:={\mathcal {J}}(\underline{\varvec{u}}^{(p-1)})$, which is equivalent to (26). We will now show that, for $\Delta t$ small enough, ${\mathcal {J}}$ is indeed a contraction. In fact, by a direct computation, it holds

36 $$\begin{aligned} \left\Vert {\mathcal {J}}(\underline{\varvec{v}})-{\mathcal {J}}(\underline{\varvec{w}})\right\Vert _\infty =\left\Vert B^{-1}\left[ \widetilde{\underline{\varvec{\phi }}}\left( \underline{\varvec{v}}\right) -\widetilde{\underline{\varvec{\phi }}}\left( \underline{\varvec{w}}\right) \right] \right\Vert _\infty \end{aligned}$$

and, applying Corollary 1 on the Lipschitz-continuity-like property of $B^{-1}\widetilde{\underline{\varvec{\phi }}}$, we retrieve the thesis

37 $$\begin{aligned} \begin{aligned} \left\Vert {\mathcal {J}}(\underline{\varvec{v}})-{\mathcal {J}}(\underline{\varvec{w}})\right\Vert _\infty = \left\Vert B^{-1}\left[ \widetilde{\underline{\varvec{\phi }}}\left( \underline{\varvec{v}}\right) -\widetilde{\underline{\varvec{\phi }}}\left( \underline{\varvec{w}}\right) \right] \right\Vert _\infty \leqslant \Delta t {\widetilde{C}}_{\text{Lip}} \left\Vert \underline{\varvec{v}}-\underline{\varvec{w}}\right\Vert _\infty \end{aligned} \end{aligned}$$

for $$\Delta t<\frac{1}{{\widetilde{C}}_{\text{Lip}}}.$$

All the local approximations, obtained by solving the nonlinear system in each control volume $C_K$, constitute a global $(M+1)$th-order accurate approximation of the analytical solution. It is piecewise polynomial in each $C_K$ and discontinuous across the faces of $C_K$ shared with other control volumes and we denote it, by an abuse of notation, as $\varvec{u}_h$.

##### Remark 4

(On the computational efficiency) In several works, the nonlinear system (24) is solved by carrying the iterative process (26) until a convergence criterion is met up to a certain tolerance [16, 34]. This leads to a waste of resources as the underlying discretization error of the system (24) with respect to the analytical solution of the PDE is of order $M+1$, and hence, smaller tolerances are in general unnecessary. In this context, it is possible to obtain an $(M+1)$th-order accurate approximation of the solution of (24) by performing exactly $M+1$ iterations. More details on this will be explained in Sect. 3.2.

#### Final Corrector Step

From the predictor step, we have in each control volume $C_K$ a local $(M+1)$th-order accurate approximation $\varvec{u}_h$ of the analytical solution in the form (22), which has been computed without considering any sort of communication between the neighboring elements. In the final corrector step, we exploit such approximation to finally get $\varvec{u}_{n+1}(\varvec{x}),$ taking into account the coupling between the elements. In particular, we consider again a weak formulation of (18) in $C_K$, but this time, we use a spatial-only test function $\varphi (\varvec{x})$ and we apply the divergence theorem in space thus getting

38 $$\begin{aligned} \begin{aligned}&\int _K \varvec{u}(\varvec{x},t_{n+1})\varphi (\varvec{x}) \text{d}\varvec{x}-\int _K \varvec{u}(\varvec{x},t_n)\varphi (\varvec{x}) \text{d}\varvec{x}\\&+ \int _{t_n}^{t_{n+1}}\!\! \int _{\partial K} \varphi (\varvec{x}) \varvec{F}(\varvec{u}(\varvec{x},t)) \cdot \varvec{\nu }(\varvec{x}) \text{d} \varvec{\sigma }\text{d}t \\&- \int _{C_K} \varvec{F}(\varvec{u}(\varvec{x},t)) \cdot \nabla _{\varvec{x}} \varphi (\varvec{x}) \text{d}\varvec{x}\text{d}t-\int _{C_K} {\varvec{S}}(\varvec{x},\varvec{u}(\varvec{x},t)) \varphi (\varvec{x}) \text{d}\varvec{x}\text{d}t=\varvec{0}, \end{aligned} \end{aligned}$$

where $\varvec{\nu }(\varvec{x})$ is the outward pointing normal to $\partial K$. The divergence theorem in space provides the desired coupling between the neighboring cells, because the solution $\varvec{u}_h$, computed locally in each control volume $C_K$ through the predictor, is discontinuous across the boundaries $\partial K$ and, thus, a numerical flux $\widehat{\varvec{F}}$ is needed to compute the flux at the cell interfaces $\partial K$. We can use either a simple and robust local Lax-Friedrichs scheme [74], or a less dissipative Osher numerical flux function [36]. At the discrete level, recalling the adopted discretization (20) for $\varvec{u}_n(\varvec{x})$ $\text{for any} \,n$, we get for each control volume $C_K$

39 $$\begin{aligned} \begin{aligned}&\sum _{i=1}^I \int _K \varphi _{i}(\varvec{x})\varphi _{j}(\varvec{x}) \text{d}\varvec{x}(\varvec{c}_i^{n+1}-\varvec{c}_i^{n}) \\&+ \int _{t_n}^{t_{n+1}} \int _{\partial K} \varphi _{j}(\varvec{x}) \widehat{\varvec{F}}(\varvec{u}_h\vert _K(\varvec{x},t),\varvec{u}_h\vert _{K^+}(\varvec{x},t)) \cdot \varvec{\nu }(\varvec{x}) \text{d} \varvec{\sigma }\text{d}t\\&- \int _{C_K} \varvec{F}(\varvec{u}_h(\varvec{x},t)) \cdot \nabla _{\varvec{x}} \varphi _{j}(\varvec{x}) \text{d}\varvec{x}\text{d}t - \int _{C_K} {\varvec{S}}(\varvec{x},\varvec{u}_h(\varvec{x},t)) \varphi _{j}(\varvec{x}) \text{d}\varvec{x}\text{d}t=\varvec{0}\end{aligned} \end{aligned}$$

for every $j=1,\cdots ,I$, with $K^+$ being the neighboring cell of *K* sharing $\partial K$ at a certain point $\varvec{x}$. Again, we remark that this step has a global character due to the computation of the numerical fluxes, but it is explicit as $\varvec{u}_h$ has been obtained in the predictor step. Let us notice that the linear systems involved in the corrector are local and even smaller than the predictor ones, thus readily invertible.

By solving the linear system (39) with respect to the coefficients $\varvec{c}_i^{n+1}$ in each element *K*, we get the final solution $\varvec{u}_{n+1}(\varvec{x})=\sum _{i=1}^I \varvec{c}_i^{n+1} \varphi _{i}(\varvec{x})$ for any $\varvec{x}\in K$ which is an $(M+1)$-th-order accurate approximation of $\varvec{u}(\varvec{x},t_{n+1}).$

### ADER-DG as DeC

It is possible to interpret the ADER-DG predictor step as a DeC procedure. We set $\Delta :=\Delta t$ and, from the local space-time nonlinear system (24), we define the high-order nonlinear operator $\mathcal {L}_{\Delta }^2:{\mathbb {R}}^{L \times Q} \rightarrow {\mathbb {R}}^{L \times Q}$ as

40 $$\begin{aligned} \mathcal {L}_{\Delta }^2(\underline{\varvec{u}}):=\underline{\varvec{u}}-B^{-1}\left[ \underline{\varvec{r}}-\widetilde{\underline{\varvec{\phi }}}(\underline{\varvec{u}})\right] . \end{aligned}$$

Since solving the operator $\mathcal {L}_{\Delta }^2$ is equivalent to solve the system (24), we have already discussed the $(M+1)$th order of accuracy of its solution.

The low-order operator $\mathcal {L}_{\Delta }^1 : {\mathbb {R}}^{L \times Q} \rightarrow {\mathbb {R}}^{L \times Q}$ is, instead, defined as

41 $$\begin{aligned} \mathcal {L}_{\Delta }^1(\underline{\varvec{u}}):=\underline{\varvec{u}}-B^{-1}\left[ \underline{\varvec{r}}-\widetilde{\underline{\varvec{\phi }}}(\underline{\varvec{u}}_0)\right] , \end{aligned}$$

with $\underline{\varvec{u}}_0$ being a vector of local space-time representation coefficients with respect to the basis $\lbrace \vartheta ^{\ell }(\varvec{x},t)\rbrace _{\ell =1,\cdots ,L}$, yielding an $O(\Delta t)$-approximation of the analytical solution in $C_K$. As an example, the vector $\underline{\varvec{u}}_0$ can be chosen, such that $\sum _{\ell =1}^L \varvec{u}_0^\ell \vartheta ^\ell (\varvec{x},t) = \varvec{u}_{n}(\varvec{x})$ for all $t \in [t_n,t_{n+1}]$. In practice, this definition is merely formal as the related terms will cancel out in the iteration and in all the needed proofs. It can be shown that the local reconstruction of the PDE solution induced by the coefficients obtained by solving $\mathcal {L}_{\Delta }^1$ is first order accurate with respect to the analytical solution. Furthermore, let us observe how the problem $\mathcal {L}_{\Delta }^1({\widetilde{\underline{\varvec{u}}}})=\underline{\varvec{z}}$ for some given $\underline{\varvec{z}}\in {\mathbb {R}}^{L \times Q}$ can be easily solved by explicitly isolating ${\widetilde{\underline{\varvec{u}}}}$.

In the following, we will prove that the operators that we have defined respect the three properties needed to apply Theorem 1, but first let us characterize the related DeC iterative procedure.

#### Iterative ADER-DG-DeC Procedure

If we characterize the iterative procedure (5) in the ADER context with the operators (41) and (40), by a direct computation, we get

42 $$\begin{aligned} \begin{aligned} \underline{\varvec{u}}^{(p)}-B^{-1}\left[ \underline{\varvec{r}}-\widetilde{\underline{\varvec{\phi }}}(\underline{\varvec{u}}_0)\right]=\,&\underline{\varvec{u}}^{(p-1)}-B^{-1}\left[ \underline{\varvec{r}}-\widetilde{\underline{\varvec{\phi }}}(\underline{\varvec{u}}_0)\right] \\&-\underline{\varvec{u}}^{(p-1)}+B^{-1}\left[ \underline{\varvec{r}}-\widetilde{\underline{\varvec{\phi }}}(\underline{\varvec{u}}^{(p-1)})\right] , \end{aligned} \end{aligned}$$

which reduces to

43 $$\begin{aligned} \underline{\varvec{u}}^{(p)}=B^{-1}\left[ \underline{\varvec{r}}-\widetilde{\underline{\varvec{\phi }}}(\underline{\varvec{u}}^{(p-1)})\right] . \end{aligned}$$

This is nothing but the fixed-point iteration (26). The advantage of having put it into a DeC formulation is that, in this context, we have at our disposal an estimate for the accuracy of $\underline{\varvec{u}}^{(p)}$ obtained at the generic iteration *p* given by (6). In particular, according to Remark 1, if $\underline{\varvec{u}}^{(0)}$ yields an $O(\Delta t)$-approximation of the analytical solution, we have that the optimal number of iterations to achieve the formal accuracy is given by $P=M+1.$ A natural choice of the initial vector is thus $\underline{\varvec{u}}^{(0)}:=\underline{\varvec{u}}_0.$

#### Proof of the Properties of the Operators $\mathcal {L}_{\Delta }^1,\mathcal {L}_{\Delta }^2$

We have that the operators $\mathcal {L}_{\Delta }^1,\mathcal {L}_{\Delta }^2$ fulfill the hypotheses of Theorem 1 as stated in the next theorem.

##### Theorem 3

(ADER-DG is DeC) The operators $\mathcal {L}_{\Delta }^1,\mathcal {L}_{\Delta }^2 : {\mathbb {R}}^{L \times Q} \rightarrow {\mathbb {R}}^{L \times Q}$, defined, respectively, in (41) and (40), fulfill the three hypotheses of Theorem 1.

##### Proof

(i) **Existence of a unique solution to** $\mathcal {L}_\Delta ^2$
  This property has been already proved in Proposition 1, since solving the operator $\mathcal {L}_{\Delta }^2$ is equivalent to solve the nonlinear system (24).
(ii) **Coercivity-like property of** $\mathcal {L}_\Delta ^1$
  We consider the infinity norm over ${\mathbb {R}}^{L \times Q}$ and two general vectors $\underline{\varvec{v}},\underline{\varvec{w}}\in {\mathbb {R}}^{L \times Q}$. The proof of this property is immediate, because, by a direct computation, we have
  44 $$\begin{aligned} \begin{aligned} \left\Vert \mathcal {L}_\Delta ^1(\underline{\varvec{v}})-\mathcal {L}_\Delta ^1(\underline{\varvec{w}})\right\Vert _\infty&=\left\Vert \underline{\varvec{v}}- \underline{\varvec{w}}\right\Vert _\infty , \end{aligned} \end{aligned}$$
  and, thus, (3) holds with $\alpha _1=1.$
(iii) **Lipschitz-continuity-like property of** $\mathcal {L}_\Delta ^1-\mathcal {L}_\Delta ^2$
  The proof of this property is based on Corollary 1. A direct computation leads to the thesis
  45 $$\begin{aligned}&\left\Vert \left[ \mathcal {L}_\Delta ^1(\underline{\varvec{v}})\!-\!\mathcal {L}_\Delta ^2(\underline{\varvec{v}})\right] \!-\!\left[ \mathcal {L}_\Delta ^1(\underline{\varvec{w}})\!-\!\mathcal {L}_\Delta ^2(\underline{\varvec{w}})\right] \right\Vert _\infty \end{aligned}$$
  46 $$\begin{aligned}&\quad =\left\Vert B^{-1}\left[ \widetilde{\underline{\varvec{\phi }}}\left( \underline{\varvec{v}}\right) -\widetilde{\underline{\varvec{\phi }}}\left( \underline{\varvec{w}}\right) \right] \right\Vert _\infty \leqslant \Delta t \widetilde{C}_{\text{Lip}} \left\Vert \underline{\varvec{v}}-\underline{\varvec{w}}\right\Vert _\infty , \end{aligned}$$
  where in (46), we applied Corollary 1.

### ADER-$\mathbb P_N \mathbb P_M$ and ADER-FV

Other formulations of ADER are available in the literature, in particular ADER-$\mathbb P_N \mathbb P_M$ [13, 33, 37, 43] is a generalization of the ADER-DG formulation. The ADER-$\mathbb P_N \mathbb P_M$ method is based on adopting, for the discretization $\varvec{u}_n(\varvec{x})$ of the solution at the time $t_n$, different local basis functions $\left\{ \lambda _{r} \right\} _{r=1,\cdots ,R}$ spanning a space of discontinuous piecewise polynomial functions of degree $N\leqslant M$, i.e., $V_N$ with $V_N:=\left\{ g \in L^2(\varOmega ) ~\text {s.t.}~ g\vert _K \in {\mathbb {P}}_N(K)\right\}$, yielding the reconstruction

47 $$\begin{aligned} \varvec{u}_n(\varvec{x}):=\sum _{r=1}^R \varvec{c}_{r}^n \lambda _{r}(\varvec{x}),\quad \forall \varvec{x}\in K. \end{aligned}$$

Then, the scheme is formally identical to the one described before. In the predictor (23), the same *M*th degree local spatial bases $\left\{ \varphi _{i}(\varvec{x}) \right\} _{i=1,\cdots ,I}$ and temporal bases $\left\{ \psi ^{m}(t) \right\} _{m=0,\cdots ,M}$ are considered, yielding a local reconstruction $\varvec{u}_h(\varvec{x},t)$ in each $C_K$ guaranteeing $(M+1)$th order of accuracy. The corrector is also identical to the one previously described (39), up to the replacement of the basis functions $\varphi _{i}$ with the basis functions $\lambda _{r}$.

The only difference with respect to the original formulation is given by the fact that, if $N<M$, a suitable *M*th-degree polynomial reconstruction $\widetilde{\varvec{u}}_{n}(\varvec{x})$ has to be considered in place of $\varvec{u}_{n}(\varvec{x})$ for the computation of the related integral over *K* in the predictor (23) to guarantee $(M+1)$th order of accuracy. Usually, $\widetilde{\varvec{u}}_{n}(\varvec{x})$ is retrieved via a WENO or CWENO reconstruction [13, 37, 43].

Let us observe that if $N=M$ and the basis $\left\{ \lambda _{r}(\varvec{x}) \right\} _{r=1,\cdots ,R}$ coincides with $\left\{ \varphi _{i}(\varvec{x}) \right\} _{i=1,\cdots ,I}$, then the ADER-$\mathbb P_N \mathbb P_M$ scheme reduces exactly to the ADER-DG previously introduced. On the other hand, the scheme obtained for $N=0$, i.e., with a piecewise constant approximation of $\varvec{u}_{n}(\varvec{x})$ over ${\overline{\varOmega }}$, is the ADER-FV scheme. One can observe that the corrector, in such a case, corresponds to an explicit $(M+1)$th-order accurate FV step. All the schemes obtained for $0<N<M$ are alternatives which vary between these two schemes.

Finally, let us notice that the predictor of such methods, being formally unchanged with respect to the original formulation, can also be seen as a DeC method in which one order is achieved at each iteration until $M+1$.

## New Efficient ADER Schemes

In this section, we will explain how to apply the novel modification to the described ADER framework. We will first introduce the efficient ADER-DG-u, obtained by simply matching the order of the space-time reconstruction in each predictor iteration with the order of accuracy achieved in the same iteration, without spoiling the original order of accuracy. Afterward, we will explain how such *p*-adaptivity strategy can be exploited to prescribe structure preservation by introducing the DOOM approach. We will focus on the ADER-DG scheme, bearing in mind that the same modifications hold true for ADER-FV and ADER-$\mathbb P_N \mathbb P_M$ schemes as well.

### Modification of ADER-DG (ADER-DG-u)

We propose to change the predictor of ADER-DG by increasing the polynomial degree of the reconstruction of the numerical solution at each iteration *p* according to the order of accuracy achieved in that specific iteration. In particular, we define for any *p* the general local basis $\lbrace \vartheta ^{\ell ,(p)}(\varvec{x},t)\rbrace _{\ell =1,\cdots ,L^{(p)}}$ given by the tensor product of space basis functions $\varphi _{i}^{(p)}(\varvec{x})$ and time basis functions $\psi ^{m,(p)}(t)$ of degree *p*. We also define the functional spaces generated by these bases as $X^{(p)}:=\left( \textrm{span}\lbrace \vartheta ^{\ell ,(p)}(\varvec{x},t) \rbrace _{\ell =1,\cdots ,L^{(p)}} \right) ^Q$.

#### Remark 5

(On the spaces $X^{(p)}$) According to our definitions of the operators (41) and (40), formally, the spaces $X^{(p)}$ in the ADER context should be spaces of coefficients of the discrete solution. However, by definition, such spaces are in bijection with the functional spaces of *Q*-dimensional polynomials whose scalar components are spanned by the bases $\lbrace \vartheta ^{\ell ,(p)}(\varvec{x},t)\rbrace _{\ell =1,\cdots ,L^{(p)}}$. Since, in this context, referring to the polynomial degree of the numerical solution in each step of the process provides a clearer overview of the method, as an abuse of notation, we denote directly $X^{(p)}$ as the functional space associated to the corresponding coefficients, bearing in mind the aforementioned bijection.

Then, the main procedure at the iteration *p* passes from the space-time representation coefficients $\underline{\varvec{u}}^{(p-1)}$ with respect to the basis of $X^{(p-1)}$, $(p-1)$th-order accurate with respect to the analytical solution, to $\underline{\varvec{u}}^{(p)}$ in $X^{(p)}$ with accuracy *p*. To perform this step, we first use an embedding ${\mathcal {E}}^{(p-1)}:X^{(p-1)}\rightarrow X^{(p)}$, for example, an interpolation or an $L^2$-projection, to pass to $\underline{\varvec{u}}^{*(p-1)} = {\mathcal {E}}^{(p-1)}(\underline{\varvec{u}}^{(p-1)}) \in X^{(p)}$. This embedding should not spoil the accuracy of the reconstructed solution.

At this point, a simple iteration of the standard method (43), with structures (25) associated with the basis $\lbrace \vartheta ^{\ell ,(p)}(\varvec{x},t)\rbrace _{\ell =1,\cdots ,L^{(p)}}$, results in $\underline{\varvec{u}}^{(p)}$ and the related *p*th-order accurate reconstruction in $C_K$. These structures read as

48 $$\begin{aligned} \left\{ \begin{aligned} \mathcal {L}_{\Delta }^{2,(p)}(\underline{\varvec{u}})&:=\underline{\varvec{u}}-\left( B^{(p)} \right) ^{-1}\left[ \underline{\varvec{r}}^{(p)}-\widetilde{\underline{\varvec{\phi }}}^{(p)}(\underline{\varvec{u}})\right] ,\\ \mathcal {L}_{\Delta }^{1,(p)}(\underline{\varvec{u}})&:=\underline{\varvec{u}}-\left( B^{(p)} \right) ^{-1}\left[ \underline{\varvec{r}}^{(p)}-\widetilde{\underline{\varvec{\phi }}}^{(p)}(\underline{\varvec{u}}_0^{(p)})\right]  \end{aligned} \right. \end{aligned}$$

with

49 $$\begin{aligned} \left\{ \begin{aligned} &B^{(p)}_{j,\ell }:=\int _K \vartheta ^{\ell ,(p)} (\varvec{x},t_{n+1})\vartheta ^{j,(p)}(\varvec{x},t_{n+1}) \text{d}\varvec{x}- \int _{C_K}\!\!\! \vartheta ^{\ell ,(p)}(\varvec{x},t) \frac{\partial }{\partial t} \vartheta ^{j,(p)}(\varvec{x},t) \text{d}\varvec{x}\text{d}t,\\ &\underline{\varvec{u}}:=\begin{pmatrix} \varvec{u}^1\\ \vdots \\ \varvec{u}^{L^{(p)}} \end{pmatrix}, \quad \underline{\varvec{r}}^{(p)} :=\begin{pmatrix} \int _K \varvec{u}_n(\varvec{x})\vartheta ^{1,(p)}(\varvec{x},t_n)\text{d}\varvec{x}\\ \vdots \\ \int _K \varvec{u}_n(\varvec{x})\vartheta ^{L^{(p)},(p)}(\varvec{x},t_n)\text{d}\varvec{x}\end{pmatrix},\\ &\widetilde{\underline{\varvec{\phi }}}^{(p)}(\underline{\varvec{u}}):=\begin{pmatrix} \int _{t_n}^{t_{n+1}} \varvec{\phi }^{(p)}_1(\underline{\varvec{u}},t) \text{d}t\\ \vdots \\ \int _{t_n}^{t_{n+1}} \varvec{\phi }^{(p)}_{L^{(p)}}(\underline{\varvec{u}},t) \text{d}t \end{pmatrix}, \end{aligned} \right. \end{aligned}$$

where $\varvec{\phi }^{(p)}_j(\underline{\varvec{u}},t):=\int _K \varvec{E}(\varvec{u}_h(\varvec{x},t),\varvec{x})\vartheta ^{j,(p)}(\varvec{x},t) \text{d}\varvec{x}$, with $\varvec{u}_h(\varvec{x},t)=\sum _{\ell =1}^{L^{(p)}}\varvec{u}^\ell \vartheta ^{\ell ,(p)}(\varvec{x},t)$ $\text{for any}\, (\varvec{x},t)\in C_K$ and $\underline{\varvec{u}}^{(p)}_0$ some local coefficients extrapolated from the initial datum $\varvec{u}_n(\varvec{x})$ in *K*, yielding an $O(\Delta t)$-approximation of the analytical solution to our PDE in $C_K$. Again, the definition of $\underline{\varvec{u}}^{(p)}_0$ is merely formal as it cancels in the iterations.

The resulting modified ADER-DG-u iterative procedure reads

50 $$\begin{aligned} \left\{ \begin{aligned}&\underline{\varvec{u}}^{(0)} = \underline{\varvec{u}}_0^{(0)},\\&{\left\{ \begin{array}{ll} \underline{\varvec{u}}^{*(p-1)} = {\mathcal {E}}^{(p-1)}\left( \underline{\varvec{u}}^{(p-1)}\right) ,\\ \underline{\varvec{u}}^{(p)}=\left( B^{(p)} \right) ^{-1}\left[ \underline{\varvec{r}}^{(p)}-\widetilde{\underline{\varvec{\phi }}}^{(p)}(\underline{\varvec{u}}^{*(p-1)})\right] , \end{array}\right. } p\geqslant 1. \end{aligned} \right. \end{aligned}$$

We remark that the superscript (*p*) in the definition of the structures in (48) and (49) is simply referred to the iteration, the modified method is still fully explicit, and all the terms at the right-hand side of the iteration formula (50) can be explicitly computed.

The accuracy evolves as follows throughout the procedure. We start with $\underline{\varvec{u}}^{(0)}$ associated with a piecewise constant $O(\Delta t)$-approximation of the solution to the PDE in $C_K$ and we perform the embedding in $X^{(1)}$ to get $\underline{\varvec{u}}^{*(0)}$, still $O(\Delta t)$-accurate. Performing the first iteration via $\mathcal {L}_{\Delta }^{1,(1)},\mathcal {L}_{\Delta }^{2,(1)}$, we get $\underline{\varvec{u}}^{(1)}$ yielding an $O(\Delta t^2)$-approximation of the solution in $C_K$. We continue iteratively with $\underline{\varvec{u}}^{(p-1)}$ associated with a $(p-1)$th-order accurate approximation of the solution in $X^{(p-1)}$ spanned by polynomial bases of degree $p-1$, that is embedded in $X^{(p)}$ obtaining $\underline{\varvec{u}}^{*(p-1)}$ with the same accuracy $p-1$. This allows to compute $\underline{\varvec{u}}^{(p)}$ via a DeC iteration with $\mathcal {L}_{\Delta }^{1,(p)},\mathcal {L}_{\Delta }^{2,(p)}$ achieving *p*th order of accuracy.

#### Remark 6

(On the accuracy of the interpolation) One must notice that the discretization in the space $X^{(p)}$, corresponding to the tensor product of polynomials of degree *p* in space and in time, allows in general a maximal order of accuracy $p+1$ with respect to the analytical solution of the PDE, corresponding to an error $O(\Delta t^{p+2})$. On the other hand, the embedding ${\mathcal {E}}^{(p)} : X^{(p)}\rightarrow X^{(p+1)}$ can be simply realized by interpolation, i.e., by evaluating the reconstruction associated with $\underline{\varvec{u}}^{(p)}\in X^{(p)}$ in the nodal values defining the $X^{(p+1)}$ basis functions, when nodal bases are employed, or by an $L^2$-projection, when more general bases are considered. In both cases, this operation can preserve at most the accuracy of order *p*, hence introducing an error of $O(\Delta t^{p+1})$. Therefore, the embedding must be performed before saturating the accuracy associated with the current polynomial basis to avoid the consequent degradation of the order.

Due to the previous remark, if the final polynomial degree of the bases in space and in time is fixed to *M*, it is convenient to perform *M* iterations in the form (50) to get $\underline{\varvec{u}}^{(M)}$, associated with the desired final discretization, plus a final iteration in the same space $X^{(M+1)}=X^{(M)}$ with the same structures as the ones used in the *M*th iteration to saturate the accuracy related to such discretization getting thus $\underline{\varvec{u}}^{(M+1)}$ yielding $(M+1)$th order of accuracy.

The degrees of the bases of the spaces $X^{(p)}$, assuming a fixed final polynomial degree equal to *M*, are summarized in Table 1 and the procedure is displayed in the following sketch:

$$\begin{aligned} \begin{aligned}&\underline{\varvec{u}}^{(0)} \xrightarrow {{\mathcal {E}}^{(0)}} & \underline{\varvec{u}}^{*(0)} \xrightarrow [\mathcal {L}_{\Delta }^{1,(1)}]{\mathcal {L}_{\Delta }^{2,(1)}} &  \underline{\varvec{u}}^{(1)} \xrightarrow {{\mathcal {E}}^{(1)}} &  \underline{\varvec{u}}^{*(1)} \xrightarrow [\mathcal {L}_{\Delta }^{1,(2)}]{\mathcal {L}_{\Delta }^{2,(2)}} &  \underline{\varvec{u}}^{(2)} \xrightarrow {{\mathcal {E}}^{(2)}} \cdots \xrightarrow [\mathcal {L}_{\Delta }^{1,(M)}]{\mathcal {L}_{\Delta }^{2,(M)}} &  \underline{\varvec{u}}^{(M)} \xrightarrow [\mathcal {L}_{\Delta }^{1,(M)}]{\mathcal {L}_{\Delta }^{2,(M)}} &  \underline{\varvec{u}}^{(M+1)}. \\&\!\!\!\!O(\Delta t) &  \!\!\!\!O(\Delta t) &  \!\!\!\!O(\Delta t^2) &  \!\!\!\!O(\Delta t^2) &  \!\!\!\!O(\Delta t^3) &  \!\!\!\!O(\Delta t^{M+1}) &  \!\!\!\!O(\Delta t^{M+2}) \end{aligned} \end{aligned}$$

**Table 1:** Increasing degrees of polynomial spaces $X^{(p)}$ varying the iteration

Space	$X^{(0)}$	$X^{(1)}$	$X^{(2)}$	$X^{(3)}$	$\cdots$	$X^{(M-1)}$	$X^{(M)}$	$X^{(M+1)}$
Polynomial degree	0	1	2	3	$\cdots$	${M-1}$	*M*	*M*

Finally, always assuming a final polynomial degree equal to *M* in the predictor, the corrector step (39) is normally performed with the $(M+1)$th-order accurate discretization given by the *M*th-degree local polynomial basis functions $\varphi _{i}^{(M)}$ used in the two last predictor iterations. This leads, in each element *K*, to the local approximation $\varvec{u}_{n+1}(\varvec{x})=\sum _{i=1}^I \varvec{c}_i^{n+1} \varphi _{i}^{(M)}(\varvec{x})$ which is $(M+1)$th-order accurate. The computational advantage of the modified method with respect to the original formulation is clear: all the iterations but the last two are performed with matrix and vector structures which are smaller, implying the solution of smaller systems. Also, the space-time discretization of $\varvec{E}(\varvec{u}_h,\varvec{x})=\textrm{div}_{\varvec{x}}{\varvec{F}}(\varvec{u}_h(\varvec{x},t))-{\varvec{S}}(\varvec{x},\varvec{u}_h(\varvec{x},t))$ and the orders of the quadrature formulas used in the low-order iterations can be suitably chosen to decrease the related computational cost. The only extra cost can come from the embedding between the spaces, which, for interpolations, can be easily recast as products by precomputable interpolation matrices, which can, therefore, be efficiently performed.

In this work, we assume modal bases in space and time. This further increases the computational advantage as, in such context, the higher order mode is easily introduced by adding zero components to $\underline{\varvec{u}}^{(p)}$, thus getting $\underline{\varvec{u}}^{*(p)}=(\underline{\varvec{u}}^{(p)},\varvec{0})^\text{T}$ without any other effort.

We denote this scheme by ADER-DG-u, referring to the $\alpha$DeCu schemes introduced in [61] with a similar technique, where u denotes the quantity that has been embedded.

#### Remark 7

(Galerkin projection) In the specific context of these new modified ADER-DG methods, the embedding procedure between the spaces $X^{(p-1)}$ and $X^{(p)}$ could be replaced by a Galerkin projection onto $X^{(p)}$. Namely, in (50), one could skip the interpolation procedure and directly consider $$\begin{aligned}\widetilde{\underline{\varvec{\phi }}}^{(p)}(\underline{\varvec{u}}^{(p-1)})\end{aligned}$$ which is defined in each *j*th component by the integral over $[t_n,t_{n+1}]$ of

51 $$\begin{aligned} \varvec{\phi }^{(p)}_j(\underline{\varvec{u}}^{(p-1)},t):=\int _K \varvec{E}(\varvec{u}^{(p-1)}_h(\varvec{x},t),\varvec{x})\vartheta ^{j,(p)}(\varvec{x},t) \text{d}\varvec{x} \end{aligned}$$

with $\varvec{u}^{(p-1)}_h(\varvec{x},t) = \sum _{\ell =1}^{L^{(p-1)}} \varvec{u}^{\ell ,(p-1)} \vartheta ^{\ell ,(p-1)}(\varvec{x},t)$. This mismatch between the spaces of the explicit term and of the test functions permits the evolution to the next space $X^{(p)}$. This is particularly convenient as there would be no interpolation, whose cost is, however, negligible with respect to the rest of the scheme. For modal bases, the two approaches are equivalent.

#### Remark 8

(Space-time accuracy) Since the ADER schemes are one-step fully discrete predictor-corrector methods on space-time control volumes $C_K$, the order of accuracy in space and time is simultaneously evolved; thus, if the iterative solution $\underline{\varvec{u}}^{(p)}$ is of order $O(\Delta t^{p+1})$ in time, it is also accurate $O(h^{p+1})$ in space, assuming a suitable CFL condition linking $\Delta t$ and *h*. This is omitted to lighten the notation.

As already remarked, since the predictor of the ADER-$\mathbb P_N \mathbb P_M$ is identical to the one of the standard ADER-DG, we can analogously introduce the ADER-$\mathbb P_N \mathbb P_M$ -u and, as a particular case, the ADER-FV-u methods in a straightforward way.

#### Remark 9

(On the memory and computational differences of ADER-DG-u) The implementation of ADER-DG and ADER-DG-u can be performed in various ways. The evolution structures of the predictor might be either pre-computed at the beginning of the simulation or on-the-fly at each time iteration (this is necessary for Lagrangian codes on moving meshes). We make use of polygonal meshes, but on triangular meshes, all operators can be pre-computed with minimal storage on a reference element.

For ADER-DG-u with respect to ADER-DG, there might be an increase in cost and storage for the different iterative structures, e.g., $B^{(p)}$, only if nodal basis functions are used. In the considered case, where modal polynomial basis functions are adopted, the iterative structures are simply constituted of slices of the highest order structures, that would be anyway computed for ADER-DG. Hence, there is no extra computation nor memory storage to be considered. In the modal case, these costs can be reduced by a pre-computation of all the operators and, in case of triangular meshes, with computations only on a reference element.

### DOOM Limiter Based on Adaptivity

In the context of the novel schemes, it is very natural to introduce a limiter that guarantees some structural properties of the solution. The limiter will be denoted by Discrete Optimally increasing Order Method (DOOM), as it will stop the iteration process in the predictor at an optimal value.

We consider the ADER-FV-u scheme, to inherit the robustness of FV formulations and the far less-restrictive CFL constraints which are suitable for large-scale simulations, and we introduce an adaptive criterion. We fix a final number of iterations $P=M+1$, corresponding to $(M+1)$th order of accuracy, and we perform the local predictor iterations as prescribed in the context of the ADER-FV-u scheme, but, in contrast with the standard method, we check for the non-violation of some physical constraints (for example the positivity of density and pressure in hydrodynamics) of the computed solution. If at iteration *p*, with $1\leqslant p\leqslant M+1$, the computed $\underline{\varvec{u}}^{(p)}$ does not fulfill some of the mentioned constraints, the solution is rejected and $\underline{\varvec{u}}^{(p-1)}$ is assumed to be the output of the iterative procedure for the correction step. Let us notice that, in the worst case, considering $\underline{\varvec{u}}^{(0)}$ in the correction step in a given region of $\varOmega$ leads to a standard first-order Godunov scheme which is, indeed, reliable. A sketch of the limiter is displayed in Algorithm 1, in which $\overline{\varvec{u}}_n$ represents the local constant value of $\varvec{u}_n(\varvec{x})$ in a cell *K* in the ADER-FV-u (and ADER-FV) context.
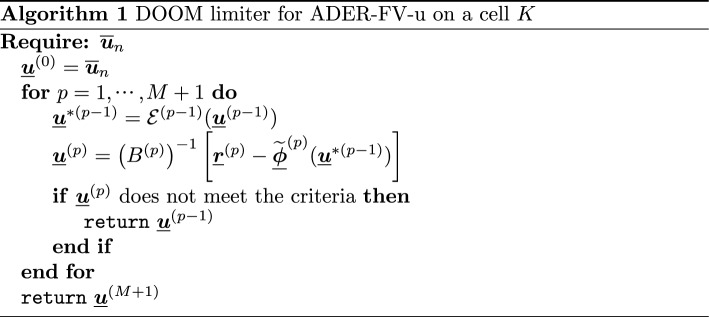


The strategy may remind the *a posteriori* MOOD technique [9, 18, 29, 31] with some fundamental differences. The low-order acceptable solution $\underline{\varvec{u}}^{(p-1)}$ has been computed before $\underline{\varvec{u}}^{(p)}$, as it was a necessary step toward the increase of an order of accuracy. Moreover, the order of accuracy is automatically pushed as much as possible without violating the physical constraints: in fact, $\underline{\varvec{u}}^{(p)}$, possibly rejected, is computed if and only if $\underline{\varvec{u}}^{(p-1)}$ was reliable. This avoids the risk of an over-diffusion in having the safe low-order scheme guaranteeing an accuracy lower than the one actually achievable. Therefore, it is then possible to preserve some physical properties through this procedure as explained in the following proposition.

#### Proposition 2

(ADER-FV-u with DOOM property) Suppose that the FV scheme preserves a property ${\mathscr {P}}$. Suppose that the property ${\mathscr {P}}$ is checked in the DOOM admissibility criteria. Then, the ADER-FV-u with the DOOM limiter preserves the property ${\mathscr {P}}$.

#### Proof

In the ADER-FV-u case, $\varvec{u}_n$ is locally represented through a single constant basis function $\lambda _1\equiv 1$. By induction on the time step *n*, suppose that the value of $\varvec{u}_n$ in each cell *K*, i.e., $\varvec{c}_{1}^n:=\overline{\varvec{u}}_n$ of (47), verifies the property ${\mathscr {P}}$. Then, independently of the WENO reconstruction of $\varvec{u}_n$ used to obtain the polynomial $\widetilde{\varvec{u}}_{n}(\varvec{x})$ for computing the related integrals in (49) with the desired accuracy, $\underline{\varvec{u}}^{(0)}=\varvec{c}_{1}^n$ still is the local value of the original $\varvec{u}_n$ and fulfills the property ${\mathscr {P}}$. Then, during the ADER-FV-u DOOM procedure, $\underline{\varvec{u}}^{(p)}$ are kept in the iterations only if property ${\mathscr {P}}$ is fulfilled. Hence, property ${\mathscr {P}}$ holds for the final predictor $\varvec{u}_h$ and, in the corrector step (39), we perform the FV method using $\varvec{u}_h$. Therefore, $\varvec{u}_{n+1}$ still fulfills property ${\mathscr {P}}$.

At the moment, this procedure does not guarantee to preserve the property for ADER-$\mathbb P_N \mathbb P_M$ with $N>0$, due to the fact that the corrector in such a case is not an explicit FV step. However, the authors are working on new structure-preserving strategies for such schemes. Moreover, in the simulations of this work, only positivity of density and pressure is checked with the DOOM limiter, but other properties like discrete local maximum principle or entropy inequalities [4, 49, 56, 57] can be ensured.

#### Remark 10

(Other applications of the *p*-adaptivity) The adaptive nature of the novel methods can be exploited also for other applications. In particular, the approach can approximate the exact solution with arbitrary precision as $p\rightarrow +\infty$ and it is not constrained to a maximum degree *M* and the related approximation accuracy. Within this framework, it is easy to design efficient arbitrary high-order adaptive schemes, as in [61] in a DeC context for ODEs, choosing the stopping criterion for the iterations in accordance with the iteration error. Also the *hp*-adaptivity can be introduced in this framework. As soon as the DOOM limiter requires low-order steps, it is possible to locally use the *h*-adaptivity to recover for the lost accuracy. These applications are already object of study of the authors, but they will not be treated in this work.

## Numerical Results

In this section, we will report the numerical results of several tests performed to validate the accuracy and the robustness of the novel ADER-DG-u and ADER-FV-u methods, i.e., ADER-$\mathbb P_N \mathbb P_M$ -u, respectively, with $N=M$ and $N=0$. To quantify the obtained speedup in terms of computational time, they will be compared with the state-of-the-art ADER-DG and ADER-FV methods [16], characterized by a fixed polynomial degree along the whole iterative procedure and a convergence criterion $\left\Vert \underline{\varvec{u}}^{(p)}-\underline{\varvec{u}}^{(p-1)}\right\Vert _\infty <{\texttt {tol}}$ to stop the predictor iterations (26), where here we assume $\texttt {tol}=10^{-12}$.

We adopt the following notation: for each method, we explicitly specify the formal order of accuracy. Therefore, ADER-DG($M+1$) and ADER-FV($M+1$) represent the original methods with predictor spatial and temporal basis functions of degree *M* guaranteeing $(M+1)$th order of accuracy. In the context of ADER-DG-u($M+1$) and ADER-FV-u($M+1$), instead, *M* is the final degree of the predictor spatial and temporal basis functions at the end of the iteration process still leading to accuracy $M+1$.

We will focus on the Euler and compressible Navier-Stokes equations. The Euler equations are a system of hyperbolic PDEs in the form (18) given by

52 $$\begin{aligned} \varvec{u}=\begin{pmatrix} \rho \\ \varvec{q}\\ E \end{pmatrix}, \quad \varvec{F}(\varvec{u}) = \begin{pmatrix} \varvec{q}\\ \rho \varvec{v}\otimes \varvec{v}+p {\mathbb {I}}\\ \varvec{v}(E+p) \end{pmatrix}, \quad \varvec{S}(\varvec{x},\varvec{u})=\varvec{0}, \end{aligned}$$

where $\rho$ is the density, $\varvec{q}\in {\mathbb {R}}^D$ the momentum, *E* the energy, *p* the pressure, $\varvec{v}=\frac{\varvec{q}}{\rho }\in {\mathbb {R}}^D$ the velocity of the flow, and ${\mathbb {I}}\in {\mathbb {R}}^{D\times D}$ is the identity matrix. The system is completed by specifying the closure equation of state $E=\frac{p}{\gamma -1} +\rho \frac{ \left\Vert \varvec{v}\right\Vert _2^2}{2}$, where $\gamma =\frac{c_p}{c_v}$ is the adiabatic coefficient defined as the ratio between the specific heats at constant pressure and volume and is here assumed to be $\gamma =1.4$.

The more general compressible Navier-Stokes equations are obtained by keeping the viscosity effects into account and, for ideal gases, are defined by

53 $$\begin{aligned} \varvec{u}=\begin{pmatrix} \rho \\ \varvec{q}\\ E \end{pmatrix}, \quad \varvec{F}(\varvec{u}) = \begin{pmatrix} \varvec{q}\\ \rho \varvec{v}\otimes \varvec{v}+\varvec{\sigma }(\varvec{u},\nabla _{\varvec{x}} \varvec{u})\\ \varvec{v}(E {\mathbb {I}} +\varvec{\sigma }(\varvec{u},\nabla _{\varvec{x}} \varvec{u}))- \kappa \nabla _{\varvec{x}} T\end{pmatrix}, \quad \varvec{S}(\varvec{x},\varvec{u})=\varvec{0}, \end{aligned}$$

where $\varvec{\sigma }(\varvec{u},\nabla _{\varvec{x}} \varvec{u})$ denotes the stress tensor, $\kappa$ is the heat conduction coefficient, and $T$ represents the temperature, while the other terms have the same meaning as in the context of the Euler equations. In particular, the stress tensor $\varvec{\sigma }(\varvec{u},\nabla _{\varvec{x}} \varvec{u})$ is given, under the Stokes hypothesis, by

54 $$\begin{aligned} \varvec{\sigma }(\varvec{u},\nabla _{\varvec{x}} \varvec{u}) = \left( p +\frac{2}{3} \mu ~ \textrm{div}_{\varvec{x}} \varvec{v}\right) {\mathbb {I}} -\mu \left( \nabla _{\varvec{x}} \varvec{v}+ \nabla _{\varvec{x}} \varvec{v}^\text{T}\right) , \end{aligned}$$

with *p* being the pressure of the fluid and $\mu$ the dynamic viscosity that we assume to be constant. The heat conduction coefficient $\kappa$ is linked to the viscosity coefficient through the Prandtl number *Pr* with the following law:

55 $$\begin{aligned} \kappa = \frac{\mu \gamma c_v}{Pr}, \end{aligned}$$

where again $\gamma = \frac{c_p}{c_v}$. A thermal and a caloric equation of state are needed for the closure of (53). For an ideal gas, those are

56 $$\begin{aligned} \frac{p}{\rho } = R T, \quad \frac{e}{\rho } = c_v T \end{aligned}$$

with *R* being the specific gas constant and $e=E-\rho \frac{\left\Vert \varvec{v}\right\Vert ^2_2}{2}$ the internal energy.

We will consider two-dimensional (2D) problems, and hence, $\varvec{v}:=(u,v)^\text{T}$.

If not stated otherwise, the CFL number is set to $\text {CFL}=0.5$, and the time step is computed according to an explicit stability condition which is given by

57 $$\begin{aligned} \Delta t\leqslant \text {CFL}\, \frac{\min \limits _{ K \in \tau _h} h_K}{(2N+1) \max \limits _{K \in \tau _h} \left( \max \limits _{\begin{array}{c} \varvec{x}\in K \end{array}} \left\Vert \varvec{\lambda }\right\Vert _\infty + 2 \max \limits _{\begin{array}{c} \varvec{x}\in K \end{array}} \left\Vert \varvec{\lambda }_v \right\Vert _\infty \frac{2N+1}{h_K} \right) }, \end{aligned}$$

where *N* represents the degree of the chosen polynomial representation, while $\varvec{\lambda }=\left( \left\Vert \varvec{v}\right\Vert _2-\sqrt{\gamma \frac{p}{\rho }},\left\Vert \varvec{v}\right\Vert _2,\left\Vert \varvec{v}\right\Vert _2+\sqrt{\gamma \frac{p}{\rho }}\right)$ are the convective eigenvalues of the Euler system, and the viscous eigenvalues $\varvec{\lambda }_v$ are given in [35]. The characteristic mesh size of the cell $h_K$ is given by the square root of its surface in two dimensions. If not stated differently, the local Lax-Friedrichs numerical flux function [74] is used in the corrector step (39).

For more challenging tests, in which the density is close to zero, we will activate the DOOM limiter checking for the positivity of the density and pressure in the quadrature points and that no NaN appears in the solution. This limiter will be used only with the ADER-FV-u technique, which provably guarantees the preservation of the positivity of these quantities.

### Numerical Convergence Studies

To test the accuracy of the method, we perform a convergence test on a smooth isentropic vortex [76] for the compressible Euler equations. The computational domain is $\varOmega = [0,10]^2$ with periodic boundary conditions, and it is tessellated by a polygonal mesh. The vortex is centered at the initial time in $\varvec{x}_c=(x_c,y_c)^\text{T}=(5,5)^\text{T}$ and moves with a background speed of $\varvec{v}_\infty =(u_\infty ,v_\infty )^\text{T}=(1,1)^\text{T}$. The initial position of the vortex, in a generic point $\varvec{x}=(x,y)^\text{T}$, can be described using the radial coordinate $r{:}=\left\Vert \varvec{x}-\varvec{x}_c\right\Vert _2$ as

58 $$\begin{aligned} {\left\{ \begin{array}{ll} \rho (\varvec{x},0) = (1+\delta T)^{\frac{1}{\gamma -1}},\\ \varvec{v}(\varvec{x},0)=\varvec{v}_\infty +\frac{\epsilon }{2\uppi }\text{e}^{\frac{1-r^2}{2}} \begin{pmatrix} -(y-y_c)\\ (x-x_c) \end{pmatrix}, \\ p(\varvec{x},0) = (1+\delta T)^{\frac{\gamma }{\gamma -1}} \end{array}\right. } \quad \delta T= -\frac{(\gamma -1)\epsilon ^2}{8\gamma \uppi }\text{e}^{1-r^2} \end{aligned}$$

with $T$ denoting the fluid temperature. The exact solution is obtained as $\varvec{u}(\varvec{x},t)=\varvec{u}(\varvec{x}-\varvec{v}_\infty t,0)$. We run the simulation until the final time $t_f=1$ using the Osher-type numerical flux function [36].

**Fig. 1:**
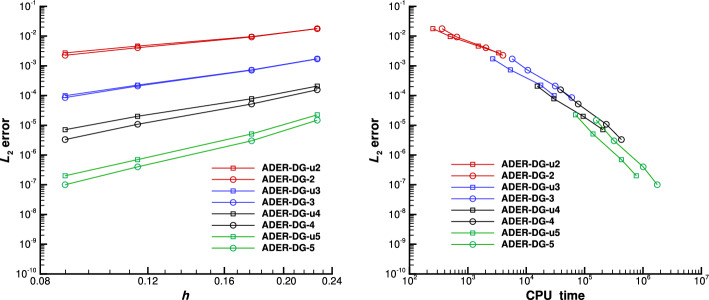
Comparison between ADER-DG-u and ADER-DG schemes from second up to fifth order of accuracy. Left: dependency of the error norm on the mesh size. Right: dependency of the error norm on the CPU time

In Fig. 1, we can observe on the left the errors of the ADER-DG and ADER-DG-u methods for different mesh sizes. All the methods achieve the formal order of accuracy. As expected, the ADER-DG-u has slightly larger errors with respect to the original ADER-DG method, as the first iterations of the predictors are done with lower order accurate operators. Nevertheless, the final error is quite comparable with the ADER-DG one and, looking at the right figure, we observe that the computational time required by ADER-DG-u for such simulations is much less (for high-order methods, it is around half) than the one required by the competitor. The slight increase in error is hugely beaten by the computational advantage of the new ADER-DG-u schemes. Indeed, the Pareto front on the right figure is only composed by ADER-DG-u points. The results are quantitatively reported in Table 2. We observe that the computed orders of accuracy are very close to the expected ones. Convergence analyses with vortex-type solutions are often subjected to some loss of order of accuracy as explained in [72, 77]. In our case, this may also be due to an imprecise choice of the mesh parameter for our polygonal meshes: we consider the maximum internal diameter of the polygons, but for some meshes, this choice might not well represent the characteristic size of the cells. The same convergence trends have been observed also in [16].

**Table 2:** Numerical convergence results for the compressible Euler equations using both ADER-DG-u and ADER-DG schemes from second up to fifth order of accuracy in space and time. The errors are measured in the $L_2$ norm and refer to the variable $\rho$ (density) at time $t_{f}=1$. The absolute CPU time of each simulation is reported in seconds

$h(\varOmega )$	ADER-DG-u			ADER-DG		
	$\rho _{L_2}$	$O(\rho _{L_2})$	CPU time/s	$\rho _{L_2}$	$O(\rho _{L_2})$	CPU time/s
	Order of accuracy: *O*(2)					
2.270E−01	1.781E−02	–	2.511E+02	1.775E−02	–	3.616E+02
1.773E−01	9.625E−03	2.49	4.997E+02	9.322E−03	2.61	6.472E+02
1.155E−01	4.614E−03	1.71	1.509E+03	4.055E−03	1.94	2.039E+03
8.786E−02	2.723E−03	1.93	3.387E+03	2.262E−03	2.14	3.989E+03

	Order of accuracy: *O*(3)					
2.270E−01	1.719E−03	–	2.664E+03	1.704E−03	–	5.750E+03
1.773E−01	7.301E−04	3.46	5.346E+03	7.121E−04	3.53	1.065E+04
1.155E−01	2.247E−04	2.75	1.773E+04	2.095E−04	2.85	3.133E+04
8.786E−02	9.871E−05	3.01	3.010E+04	8.542E−05	3.29	6.020E+04

	Order of accuracy: *O*(4)					
2.270E−01	2.076E−04	–	1.547E+04	1.563E−04	–	3.868E+04
1.773E−01	7.803E−05	3.96	2.975E+04	5.195E−05	4.46	7.766E+04
1.155E−01	2.013E−05	3.16	9.427E+04	1.085E−05	3.65	2.354E+05
8.786E−02	7.139E−06	3.80	2.054E+05	3.332E−06	4.32	4.270E+05

	Order of accuracy: *O*(5)					
2.270E−01	2.238E−05	–	6.993E+04	1.475E−05	–	3.171E+05
1.773E−01	5.080E−06	6.00	1.393E+05	3.002E−06	6.44	6.390E+05
1.155E−01	7.405E−07	4.49	4.261E+05	4.180E−07	4.60	2.015E+06
8.786E−02	2.154E−07	4.52	7.691E+05	1.228E−07	4.48	3.516E+06

**Fig. 2:**
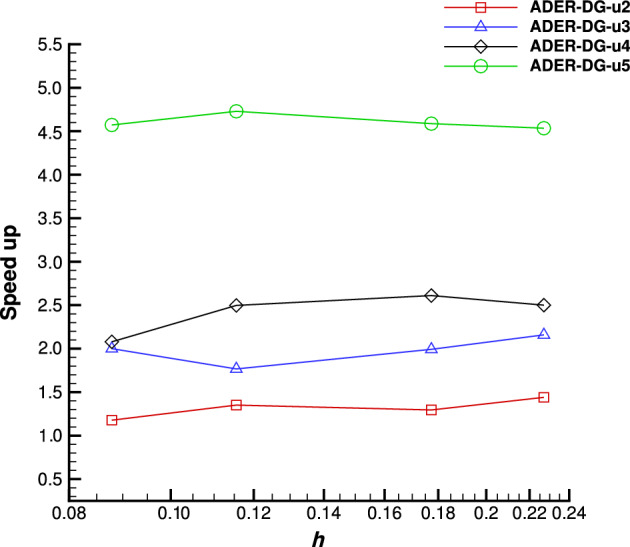
Speedup of the ADER-DG-u schemes compared to the ADER-DG methods depending on the mesh size for different orders

Finally, Fig. 2 depicts the speedup achieved by the novel adaptive schemes compared against the classical formulation of iterative methods, namely ADER-DG-u versus ADER-DG. As the order of accuracy increases, the speedup becomes higher obtaining efficient schemes which are up to $\approx 4.5$ times faster than the classical methods. Let us notice that the formal order of accuracy is still maintained, while getting a remarkable gain in the computational efficiency.

### Riemann Problems

In this section, we will show the results of the ADER-FV-u4 scheme, i.e., with $M=3$, for some Riemann problems. The computational domain is the box $\varOmega = [-0.5,0.5]\times [-0.05,0.05]$ with periodic boundary conditions in the *y* direction and Dirichlet boundaries imposed at $x=\pm 0.5$. We use an unstructured polygonal mesh made of $N_h=2\,226$ control volumes of characteristic mesh size of $h\approx \frac{1}{100}$. Despite the one-dimensional setting of the test case, we underline that the preservation of symmetry of the solution is not trivial on unstructured meshes, where no cell boundaries are in principle aligned with the main flow velocity. We solve again the Euler equations (52) with the initial conditions given, as a function of the *x* coordinate only, by

59 $$\begin{aligned} \varvec{u}(x,0)={\left\{ \begin{array}{ll} \varvec{u}_L, &{} \text {if }x<0,\\ \varvec{u}_R, &{} \text {else}, \end{array}\right. } \end{aligned}$$

where the values of $\varvec{u}_L$ and $\varvec{u}_R$ and the final times for the different tests are taken from [80] and they can be found in Table 3. The velocity along the *y*-direction is set to be $v=0$ for all the tests.

The DOOM limiter is here active checking for the positivity of density and pressure and avoiding NaN. These tests are very challenging and not all the numerical methods can stably perform on them. In particular, shocks are often not well captured or numerical oscillations appear around them and it is common that negative density or pressure values appear in the simulations, making the code crash.

**Table 3:** Initial conditions for Riemann problems

Test	$\rho _L$	$u_L$	$p_L$	$\rho _R$	$u_R$	$p_R$	$t_f$
1	0.445	0.698	3.528	0.5	0	0.571	0.14
2	1	2	0.1	1	−2	0.1	0.8
3	1	−2	0.4	1	2	0.4	0.15
4	1	0	1 000	1	0	100	0.012

**Fig. 3:**
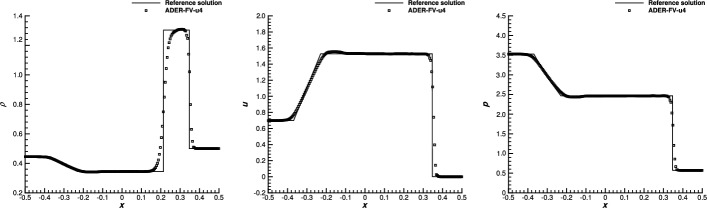
Lax shock tube problem (RP1) at the final time $t_f=0.14$. Comparison of density, velocity, and pressure versus the reference solution for the ADER-FV-u4 scheme

The first test (RP1) is the classical Lax shock tube problem. The initial discontinuity develops into a rarefaction wave, a contact discontinuity, and a shock. In Fig. 3, we observe that the ADER-FV-u does not exhibit any oscillations around the shock and that exactly catches the speed of the discontinuities.

**Fig. 4:**
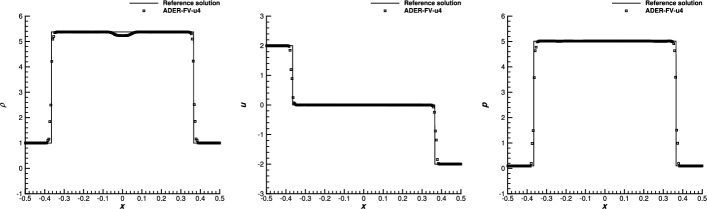
Colliding shock test (RP2) at the final time $t_f=0.8$. Comparison of density, velocity, and pressure versus the reference solution for the ADER-FV-u4 scheme

The second test (RP2) consists of a colliding shock test. The initial discontinuity in the velocity gives rise to two shocks traveling outside the domain. This test creates a very high density and pressure region in the middle of the domain. As it can be seen in Fig. 4, the new ADER-FV-u with DOOM limiter is able to perfectly capture the shock behavior within few cells without over/under-shootings at the sides of the shocks.

**Fig. 5:**
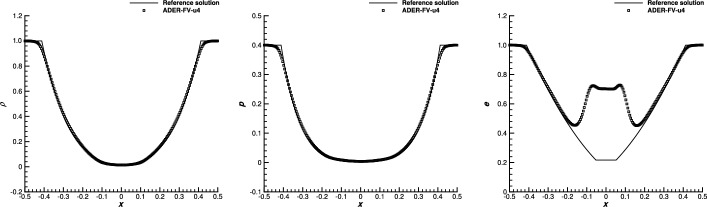
Double rarefaction test (RP3) at the final time $t_f=0.15$. Comparison of density, pressure, and internal energy versus the reference solution for the ADER-FV-u4 scheme

The next problem (RP3) is the one presented as Test 2 in [80, Section 4.3.3]. It is a double rarefaction waves which leads to very low pressure and density areas at the center of the domain. In Fig. 5, we can appreciate the capability of the scheme of maintaining positive quantities for these variables, thanks to the DOOM limiter which, at the beginning of the simulation, ensures positivity preservation in the predictor. The mismatching of the internal energy distribution is essentially due to the excessive numerical dissipation of the scheme, which could be reduced by introducing entropy preserving techniques [23, 44, 57].

**Fig. 6:**
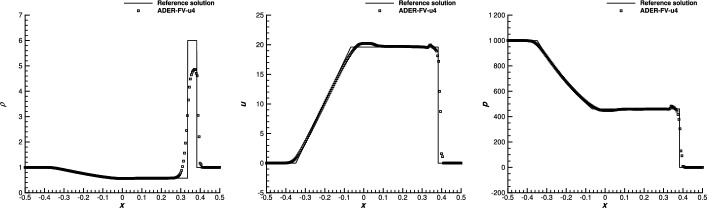
Test RP4 at the final time $t_f=0.012$. Comparison of velocity and pressure versus the reference solution for the ADER-FV-u4 scheme

The last Riemann problem (RP4) is the one presented as Test 3 in [80, Section 4.3.3]. It is a very severe test problem, and it consists of a rarefaction, a contact discontinuity, and a shock. The results obtained in Fig. 6 are in agreement with the reference solution and the smearing around the contact discontinuity is comparable to other high-order FV schemes with similar resolution.

### Viscous Shock Profile

Now, we consider an isolated viscous shock that is traveling through a medium at rest with a shock Mach number $M_s>1$ [11, 15, 16, 20, 33]; thus, we solve the compressible Navier-Stokes equations (53). The analytical solution and the details to compute it can be found in [11], where the stationary shock wave at a Prandtl number $Pr=0.75$ is resolved with constant viscosity. The computational domain is $\varOmega = [0,1]\times [0,0.2]$, which is discretized by $N_h=1\,120$ Voronoi elements. On the left side of the domain, a constant inflow velocity is prescribed, while outflow boundary conditions are assumed at the right of the domain. Periodic boundary conditions are, instead, assigned to the top/bottom boundaries. The initial condition consists of a shock wave centered at $x=0.25$ propagating at Mach $M_s = 2$ from left to right with a Reynolds number $Re = 100$; thus, the viscosity coefficient is set to $\mu = 2 \cdot 10^{-2}$. The upstream shock state is defined, such that the adiabatic sound speed is $c_0=1$. The final time of the simulation is $t_f = 0.2$ with the shock front located at $x = 0.65$.

**Fig. 7:**
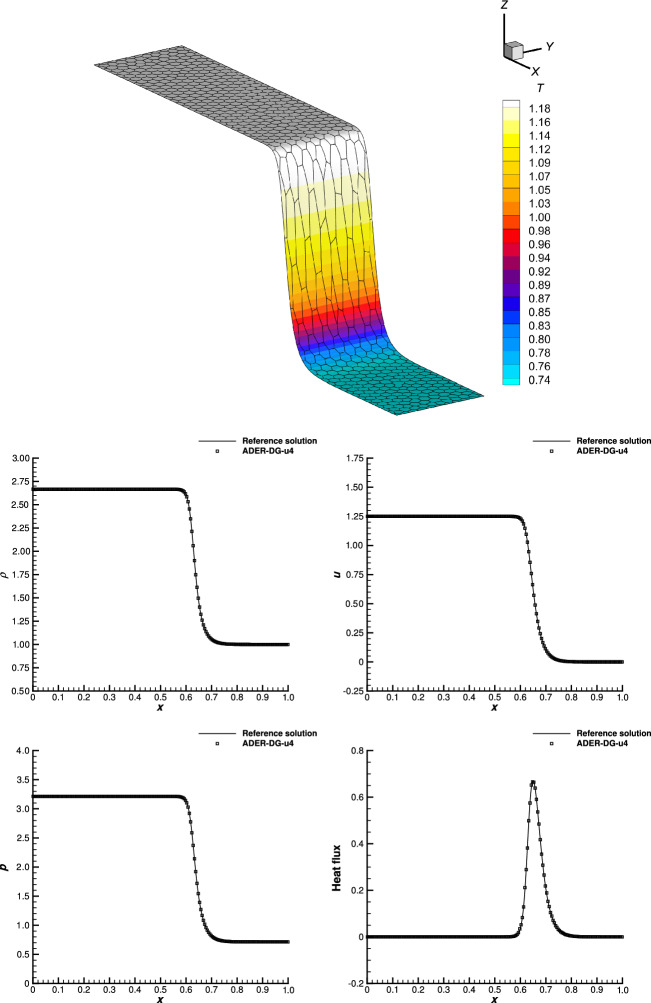
Viscous shock profile with a shock Mach number $M_s=2$ and a Prandtl number $Pr=0.75$ at time $t_f=0.2$. Top panel: Voronoi tessellation and temperature distribution along the $z$-axis. The fourth-order numerical solution with the ADER-DG-u scheme compared against the reference solution for the density, horizontal velocity, pressure, and heat flux (from middle left to bottom right panel): in particular, we show a one-dimensional cut of 200 equidistant points along the $x$-direction at $y=0.1$

We run the simulations with ADER-DG-u(4). Since the solution is smooth, nothing is checked along the DOOM procedure. Qualitatively, we see in Fig. 7 that there is an excellent agreement between the numerical solution and the analytical one. We underline that this test case allows all terms contained in the Navier-Stokes system to be properly checked, since advection, thermal conduction, and viscous stresses are present.

### 2D Taylor-Green Vortex

**Fig. 8:**
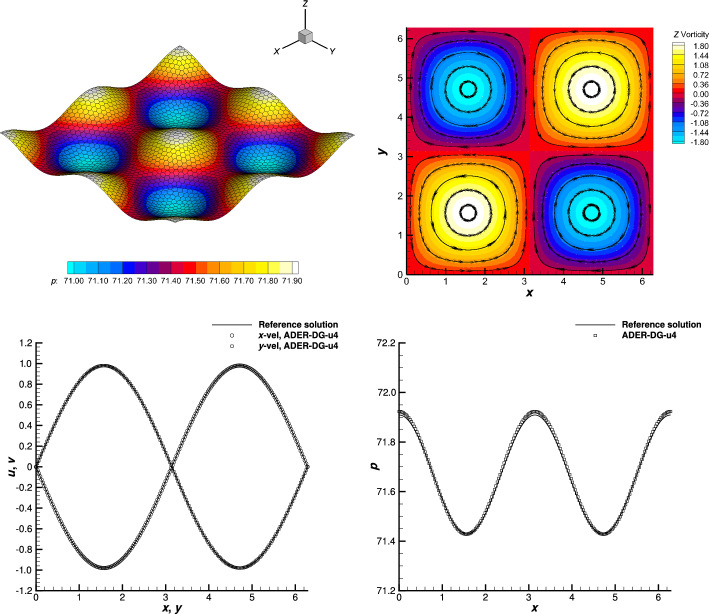
2D Taylor-Green vortex at time $t_f=1$ with viscosity $\mu =10^{-2}$. The exact solution of the Navier-Stokes equations and the fourth-order numerical solution with the ADER-DG-u4 scheme. Top: mesh configuration with pressure distribution (left) and $z$-vorticity with stream traces (right). Bottom: one-dimensional cut of 200 equidistant points along the *x*-axis and the $y$-axis for the velocity components *u* and *v* (left) and for the pressure *p* (right)

A classical test case for the incompressible Navier-Stokes equations is the Taylor-Green vortex problem. In two dimensions, the exact solution is known and it is given on the domain $\varOmega = [0,2\uppi ]^2$ with periodic boundary conditions by

60 $$\begin{aligned} \left\{ \begin{aligned} & u(\varvec{x},t) =\sin (x)\cos (y) \text{e}^{-2\nu t},\\ & v(\varvec{x},t) = -\cos (x)\sin (y) \text{e}^{-2\nu t},\\ & p(\varvec{x},t) = C+\frac{1}{4} (\cos (2x)\cos (2y))\text{e}^{-4\nu t} \end{aligned} \right. \end{aligned}$$

with $\nu = \frac{\mu }{\rho }$ the kinematic viscosity and $\mu =10^{-2}$. In this test, we also validate the quality of the scheme in a low Mach regime. Hence, the additive constant for the pressure is chosen as $C=\frac{100}{\gamma}$ and the density is set at the beginning as $\rho (\varvec{x},0)\equiv 1$. For this test, heat conduction is neglected, i.e., $\kappa =0$. The mesh is discretized by $N_h=2\,916$ cells and the final time is set at $t_f=1$. We use the ADER-DG-u method with order 4 for this simulation without checks in the DOOM procedure. The results are depicted in Fig. 8, which are compared against the analytical solutions, obtaining an excellent matching.

We also compare the numerical results with the ADER-DG scheme, and the errors are reported in Table 4 as well as the computational time. We observe that the errors are almost the same for both methods, while the novel ADER-DG-u scheme is 2.5 times faster than the classical ADER-DG.

**Table 4:** Error analysis for the Taylor-Green vortex using both ADER-DG-u and ADER-DG schemes with fourth order of accuracy in space and time. The errors are measured in $L_2$ and $L_{\infty }$ norms and refer to the variables $\rho$ (density) and horizontal velocity *u* at the final time $t_{f}=1$. The computational time measured in seconds is also reported

Scheme	Density ($\rho$)		Velocity (*u*)		CPU time
	$L_2$	$L_{\infty }$	$L_2$	$L_{\infty }$	/s
ADER-DG-u4	8.950E−03	3.305E−03	1.604E−03	6.112E−04	1.154E+04
ADER-DG4	8.950E−03	3.305E−03	1.604E−03	6.112E−04	2.706E+04

### Compressible Mixing Layer

Finally, we test the novel ADER-DG-u4 on the unsteady compressible mixing layer studied in [30]. The 2D computational domain is the rectangular box $\varOmega =[-200,200] \times [-50,50]$, and a total number of $N_h=15\,723$ polygonal Voronoi cells compose the computational mesh. The initial condition of the flow is given by two fluid layers moving with different velocities along the $x$-direction, that is

61 $$\begin{aligned} \left\{ \begin{aligned} \rho (\varvec{x},0)= & {}\, \rho _0= 1, \nonumber \\ \varvec{v}(\varvec{x},0)= & {} \, \varvec{v}_0 = \left( \begin{array}{c} \frac{1}{8} \tanh (2y) + \frac{3}{8} \\ 0 \end{array} \right) , \\ p(\varvec{x},0)= & {} \, p_0 = \frac{1}{\gamma }. \nonumber \end{aligned} \right. \end{aligned}$$

The free stream velocities are imposed as boundary conditions in the $y$-direction, and thus, we set $u_{+\infty }=0.5$ and $u_{-\infty }=0.25$ for $y \rightarrow + \infty$ and $y \rightarrow -\infty$, respectively. Along the $x$-direction, the right side is simply assigned an outflow boundary, whereas the left side is given a time-dependent inflow boundary condition with a perturbation $\delta (y,t)$

62 $$\begin{aligned} \left\{ \begin{aligned}& \rho (0,y,t)=  {} \rho _0 + 0.05 \, \delta (y,t), \nonumber \\ & \varvec{v}(0,y,t)=  {} \varvec{v}_0 + \left( \begin{array}{c} 1.0 \\ 0.6 \end{array} \right) \, \delta (y,t), \\&  p(0,y,t)=  {} p_0 + 0.2 \, \delta (y,t). \nonumber \end{aligned} \right. \end{aligned}$$

**Fig. 9:**
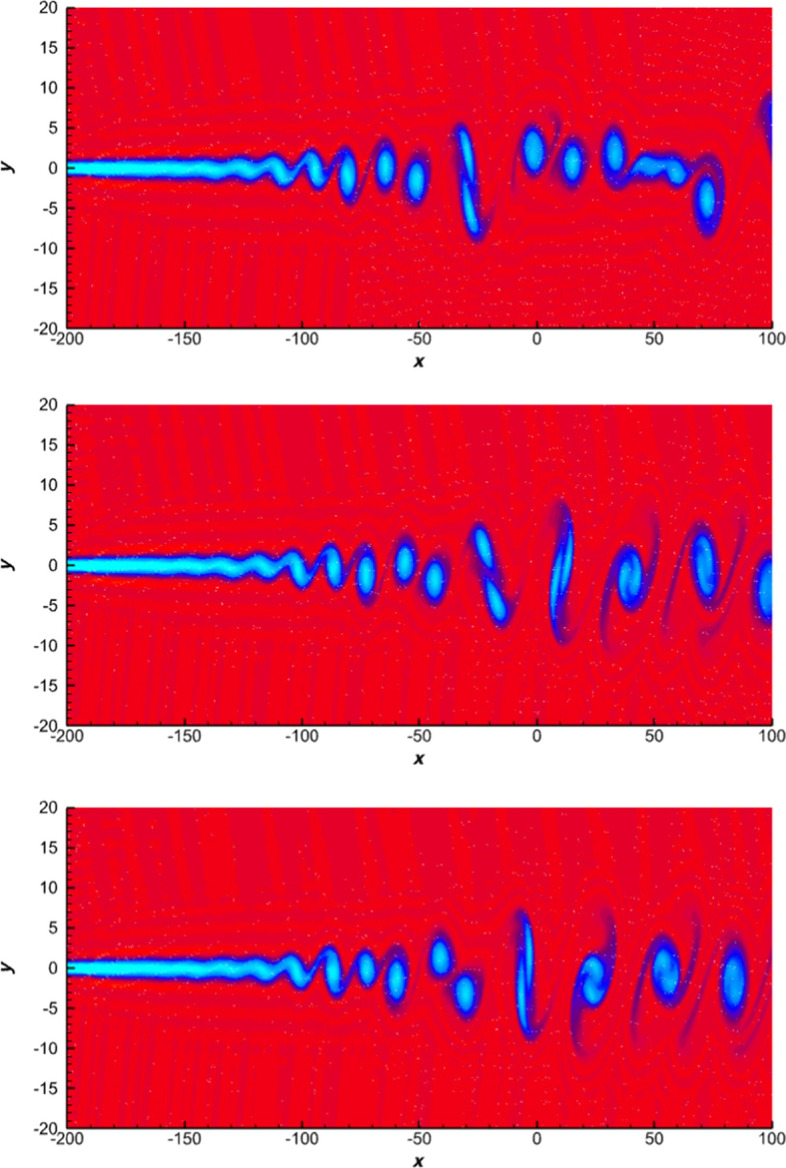
Compressible mixing layer at time $t=500$, $t=1\,000$, and $t=1\,596.8$ (from top to bottom row). Fourth-order numerical results with ADER-DG-u for $z$-vorticity. Fifty-one contour levels in the range $[-0.12,0.12]$ have been used for plotting the vorticity distribution on the sub-domain $[-200,100]\times [-20,20]$

The function $\delta (y,t)$ is given by

63 $$\begin{aligned} \delta (y,t)= & -10^{-3} \exp (-0.25 y^2) \cdot \nonumber \left[ \cos (\omega t) + \cos \left( \frac{1}{2}\omega t -0.028\right) + \cos \left( \frac{1}{4}\omega t +0.141\right) + \cos \left( \frac{1}{8}\omega t +0.391\right) \right] \end{aligned}$$

with the fundamental frequency of the mixing layer $\omega = 0.314\,787\,6$. The compressible Navier-Stokes equations are considered with the viscosity coefficient $\mu =10^{-3}$ and no heat conduction ($\kappa =0$). The final time is $t_f=1\,596.8$ and the DG solution is depicted in Fig. 9 at three different output times. The vorticity of the flow field is shown, demonstrating the capability of the novel methods to capture the complex vortical structures generated by the perturbation assigned at the inflow of the channel.

## Conclusions and Further Developments

To sum up, generalizing the idea proposed in [61], we have introduced a new framework for the construction of efficient *p*-adaptive arbitrary high-order methods, based on the modification of underlying arbitrary high-order iterative schemes. Specifically, the accuracy of the discretization is progressively increased with the number of iterations, gaining one order of accuracy at each iteration. Given an implementation of an iterative arbitrary high-order method, the novel technique is easy to include and it gives a remarkable advantage in terms of computational costs. Moreover, in this context, the *p*-adaptivity can be achieved very naturally inserting some criteria to stop the iterations. We showed an application to ADER-DG, designing the new efficient ADER-$\mathbb P_N \mathbb P_M$ -u methods. In particular, in the ADER-FV-u context ($N=0$), we have proposed DOOM, an *a posteriori* limiter, that is able to preserve the physical properties of the solution (i.e., positivity of density and pressure) obtaining the maximum admissible order of accuracy that guarantees these physical constraints to be respected. In this framework, there is a huge advantage with respect to similar *a posteriori* limiters, e.g., MOOD [29], as DOOM is waste-free, i.e., all the computations are useful either for increasing the order of accuracy or for detecting a troubled state. In the numerical tests, we have solved Euler and compressible Navier-Stokes equations very robustly, provably keeping the positivity of density and pressure, and with computational costs up to four times smaller than the original method.

We believe that the proposed framework is very versatile and can improve many arbitrary high-order methods on different sides: reducing the computational costs, easily providing the *p*-adaptivity in a very efficient and natural way without wasting computed solutions, and helping obtaining structure-preserving solutions. The authors are currently working on the application of the novel framework to obtain: adaptive methods that converge to the analytical solution up to a given tolerance and *hp*-adaptive methods introducing local mesh refinements in non-smooth regions. We also aim at further investigations on the implicit version of the schemes obtained by choosing a low order implicit operator $\mathcal {L}_{\Delta }^1$ in the DeC formulation and on structure-preserving.

## Appendix A DG Modal Taylor Basis Functions

The basis functions $\left\{ \vartheta ^{\ell }(\varvec{x},t) \right\} _{\ell =1,\cdots ,L}$ used to span the predictor polynomial spaces in this work are modal Taylor basis functions. As already said, they are the tensor products of space basis functions $\left\{ \varphi _{i}(\varvec{x}) \right\} _{i=1,\cdots ,I}$ and time basis functions $\left\{ \psi ^{m}(t) \right\} _{m=0,\cdots ,M}.$

The spatial basis functions $\left\{ \varphi _{i}(\varvec{x}) \right\} _{i=1,\cdots ,I}$ of degree at most *M* in *D* dimensions are defined locally for each element *K*. Denoting by $\varvec{x}_K$ the barycenter of the element, they can be defined as

A1 $$\begin{aligned} \varphi _{\alpha } (\varvec{x}):= \prod _{d=1}^{D} \frac{(x_d-x_{K,d})^{\alpha _d}}{\alpha _d! h_K^{\alpha _d}},\quad 0\leqslant \left|\alpha \right| \leqslant M, \end{aligned}$$

where $h_K=\root D \of {\left|K\right|}$ is the characteristic mesh size of the element *K*, used to rescale the functions to agree with the Taylor expansion terms, while $\alpha$ is a *D*-dimensional multi-index with $\left|\alpha \right|=\sum \limits_{d=1}^D\alpha _d$. In practice, we identify the multi-index $\alpha$ as a single index $i=1,\cdots ,I$ with $I={{M+D}\atopwithdelims (){D}}$ via a bijection giving $i = i(\alpha )$.

The time basis functions $\left\{ \psi ^{m}(t) \right\} _{m=0,\cdots ,M}$ of degree at most *M* are defined in a similar fashion, but with respect to a scalar argument only, over $[t_{n},t_{n+1}]$

A2 $$\begin{aligned} \psi ^{m} (t):= \frac{(t-t_{n})^{m}}{m! \Delta t^{m}},\quad 0\leqslant m \leqslant M. \end{aligned}$$

Finally, the tensor product between the two functional spaces gives the space-time basis functions $\lbrace \vartheta ^{\ell }(\varvec{x},t)\rbrace _{\ell =1,\cdots ,L}$. For full reproducibility, we specify that accuracy $M+1$ has been achieved selecting space-time basis functions up to degree *M* only. Also, for the spaces $\lbrace \vartheta ^{\ell ,(p)}(\varvec{x},t)\rbrace _{\ell =1,\cdots ,L^{(p)}}$, we have used basis functions up to degree *p* only.

### Remark A1

(On the ordering of the space-time basis functions) The novel approach is based on the adoption of iteration-specific bases $\lbrace \vartheta ^{\ell ,(p)}(\varvec{x},t)\rbrace _{\ell =1,\cdots ,L^{(p)}}$ of increasing degree. For modal bases, the introduction of higher order modes is simply performed by considering higher order terms in the space-time polynomial expansion. Therefore, in the context of an efficient implementation of the new methods, it is particularly useful to directly define all the basis functions $\lbrace \vartheta ^{\ell ,(M)}(\varvec{x},t)\rbrace _{\ell =1,\cdots ,L^{(M)}}$, up to an accuracy order $M+1$, ordering them in an increasing polynomial order. By doing so, it is enough to change the final index from $L^{(p-1)}$ to $L^{(p)}$ to pass from $X^{(p-1)}$ to $X^{(p)}$ in all the iterations but the last one, which is performed without changing polynomial space to saturate the related accuracy.
